# Integrated Taxonomy Reveals Hidden Diversity in Northern Australian Fishes: A New Species of Seamoth (Genus *Pegasus*)

**DOI:** 10.1371/journal.pone.0149415

**Published:** 2016-03-02

**Authors:** Deborah Osterhage, John J. Pogonoski, Sharon A. Appleyard, William T. White

**Affiliations:** Australian National Fish Collection, National Research Collections Australia, Commonwealth Scientific and Industrial Research Organisation, Hobart, Tasmania, Australia; Laboratoire de Biologie du Développement de Villefranche-sur-Mer, FRANCE

## Abstract

Fishes are one of the most intensively studied marine taxonomic groups yet cryptic species are still being discovered. An integrated taxonomic approach is used herein to delineate and describe a new cryptic seamoth (genus *Pegasus*) from what was previously a wide-ranging species. Preliminary mitochondrial DNA barcoding indicated possible speciation in *Pegasus volitans* specimens collected in surveys of the Torres Strait and Great Barrier Reef off Queensland in Australia. Morphological and meristic investigations found key differences in a number of characters between *P*. *volitans* and the new species, *P*. *tetrabelos*. Further mt DNA barcoding of both the *COI* and the slower mutating *16S* genes of additional specimens provided strong support for two separate species. *Pegasus tetrabelos* and *P*. *volitans* are sympatric in northern Australia and were frequently caught together in trawls at the same depths.

## Introduction

New species are not only discovered through surveys of unexplored places, but also through taxonomic studies of known species that uncover previously unknown, hidden diversity. Identifying species complexes can be a difficult process as their superficial morphology is often very similar, but failure to delineate cryptic species may confound our understanding of evolutionary and ecological processes [1]. Furthermore, resolution of species complexes often results in a single wide-ranging species being split into two or more species usually with more restricted distribution, which may have significant management implications [2]. In a world of increasing environmental impacts, uncovering the hidden diversity around us is of growing importance.

Cryptic species are common in the marine environment and fish are one of the most intensively studied marine taxonomic groups, yet traditional taxonomic methods often fail to identify cryptic fish species [1,3]. Morphological variation, such as sexual dimorphism, is common within fish species and may lead to difficulty in separating intraspecific or interspecific differences among cryptic species that display almost identical external morphology. DNA barcoding, typically using the cytochrome oxidase subunit 1 (*COI*) gene, is an extremely useful tool for delineating fish species and has proven effective at highlighting cryptic speciation [4,5,6]. The ‘barcoding gap’, referred to by [7], is the difference in DNA sequences between individuals within a species versus those among species. DNA barcoding and sequence comparisons of unknown specimens to previously documented and vouchered specimens in annotated publicly available international databases such as BOLD (http://www.barcodinglife.com/) and GenBank (http://www.ncbi.nlm.nih.gov/genbank/) is widely accepted as part of an integrated methodology (combined with traditional taxonomic methods) for facilitating the rapid species identification of unidentified species and resolution of species complexes. Integrated taxonomy methodologies as suggested in this study, utilises multiple lines of evidence to provide rigour to species delineation and has been used to effectively identify, delineate, and describe cryptic fish species and other taxa in the marine environment [6,8,9,10].

*Pegasus volitans*, or the slender seamoth, is a small, benthic fish that is listed as data deficient by the IUCN’s *Red List of Threatened Species* [11]. They are caught as bycatch in trawl fisheries throughout their range and in some areas are utilised both alive as an aquarium species, and dead and dried for traditional Chinese medicine [12]. Between 2003 and 2005, a large number of *Pegasus volitans* were collected in scientific surveys of the Torres Strait and Great Barrier Reef in Queensland, Australia. Preliminary DNA barcoding of the specimens unexpectedly revealed possible cryptic speciation within *P*. *volitans*. COI divergence of greater than 2% has been found to indicate speciation for over 1000 fish species [13], and the resulting two genetic groups of *P*. *volitans* specimens suggested that a species complex existed. To substantiate this result, an integrated taxonomic approach was employed: barcoding both the *COI* and the slower mutating 16S rRNA (*16S*) genes of additional specimens, as well as using classical taxonomic techniques with the aim of providing multiple supporting evidence to delineate and ultimately describe a new *Pegasus* species.

### Taxonomic overview

The family Pegasidae (Syngnathiformes) contains 5 valid species within 2 genera, *Pegasus* and *Eurypegasus*, which are distributed throughout the Indo-West & Central Pacific [14]. The genus *Pegasus* consists of 3 currently recognised species, *P*. *lancifer* Kaup, *P*. *laternarius* Cuvier and *P*. *volitans* Linnaeus. The two latter species have an Indo-West Pacific distribution, whilst *P*. *lancifer* is endemic to southern Australia. *Pegasus volitans* is currently recognised as having the broadest range, i.e. from East Africa to Australia and southern Japan, and is the only *Pegasus* species known to occur in Queensland waters.

*Pegasus volitans* was first proposed by [15] based on 3 literature sources, [16], [17], and on an unpublished work of his own, *Museum Adolphi Friderici* Volume 2, later published as [18]. The account of *Pisciculus amboinensis volans*, *osseo-tuberculosus*, *proboscides errata* by [17], also references the [16] record. The account in [17] was related by [19] to specimen ZMUC P8430 in the Københavns Universitet, Zoologisk Museum in Copenhagen. This specimen is missing the caudal fin, as is the specimen illustrated by [16], which provides further evidence they were based on the same specimen. The third source, Linnaeus’s own unpublished work and later published as [18], has been linked directly to a specimen deposited in the Naturhistoriska Riksmuseet in Stockholm, NRM LP 30, by [20]. The location provided by [15] for this species was Amboina (= Ambon, Indonesia) based on the [16] and [17] records.

**Fig 1:**
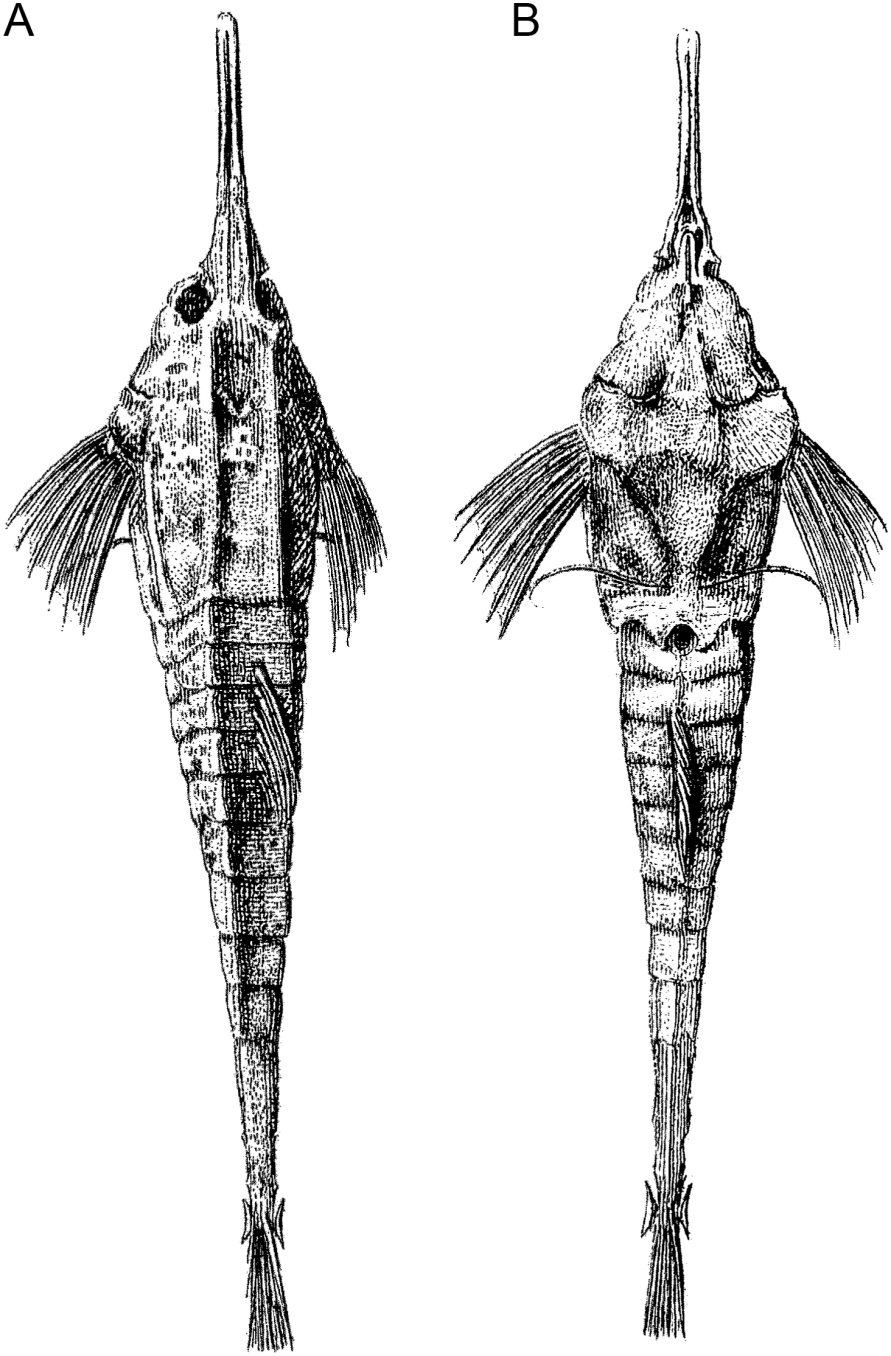
*Cataphractus corpora oblong plagioplateo* illustration. (A) dorsal; and (B) ventral view of the record upon which *Pegasus natans* was solely based [21].

The taxonomy of this family quickly became complicated when, after realising the composite nature of his original 1758 description, [18] proposed 3 new species within *Pegasus*: *P*. *draconis* based on the [16] and [17] records, ZMUC P8430; *P*. *volans* from his account of *P*. *volitans* in [18] based on the NRM LP 30 specimen; and *Pegasus natans* based on [21]. *Pegasus draconis* has since been allocated to the genus *Eurypegasus*. Since *P*. *volans* was based on the same record as Linnaeus’s unpublished work which formed the primary basis for *P*. *volitans* [15], *P*. *volans* is a junior synonym of *P*. *volitans* and the specimen NRM LP 30 stands as the holotype for the former and syntype (herein lectotype) of the latter nominal species (see [20]). *Pegasus natans* was based solely on ‘Cataphractus corpora oblong plagioplateo’ in [21], which consists of a relatively detailed description with both dorsal and ventral illustrations (Fig 1). No type specimens exist for *P*. *natans* [22]. The location given by [21] was *Indiis*, probably in reference to modern day Indonesia. This species is considered by most to be a synonym of *P*. *volitans*, based on the illustrations and description by [21] (see [14]). *Pegasus natans* and *P*. *volans* were differentiated by [23] by the rostrums, with *P*. *volans*’ rostrum described as denticular and that of *P*. *natans* as ‘unarmed’. This is unusual as, although [21] does not stipulate the denticular nature of the rostrum, he likewise does not describe it as otherwise. Without a type specimen related to the [21] description, it is impossible to qualify this difference.

*Pegasus pristis* was described by [24] based on 5 specimens collected from Padang in Sumatra (Indonesia). However, Bleeker later synonymises *P*. *pristis* under *P*. *natans* citing insufficient descriptions by previous authors as the cause of this confusion [25]. *Pegasus pristis* is thus considered a synonym of *P*. *volitans*, since *P*. *natans* is currently considered as such. The name *Cataphractus anceps* from ‘*Mari Indico*’ (= Indian sea) was proposed by [26] based on the record of [21], and includes Linnaeus’ *P*. *natans* in its synonymy. As it is based on the same source as that used by [23] for *P*. *natans* which is currently considered a synonym of *P*. *volitans*, *C*. *anceps* is a junior synonym of *P*. *volitans*. There are no known types of *C*. *anceps* [15].

## Materials and Methods

### Ethics Statement

Most of the specimens of the new species and *Pegasus volitans* from Queensland were obtained during two extensive scientific projects in the Torres Strait and Great Barrier Reef, Australia between 2003 and 2005 [27,28]. In both regions, specimens were collected by trawling. A single high-flying Florida Flyer net with a head rope length of 14.6 m and stretched mesh size of 50 mm of 4040D/27 ply was towed over the stern of the *FRV Gwendoline May*. The net was towed in a relatively straight line for 1 km at a speed of about 2.7 knots. All trawling was conducted between an hour after sunset and dawn [27,28].

No animal ethics approval was required to undertake this trawling in Queensland waters during the study period (2003–2005). A permit to undertake scientific trawling and collection of specimens was obtained from the Great Barrier Reef Marine Park Authority (GRMBA; permit number G03/7584.1). We obtained permission from the Australian Museum in Sydney (AMS), Australian National Fish Collection in Hobart (CSIRO), the Museum and Art Gallery of the Northern Territory (NTM), the Queensland Museum in Brisbane (QM), and the Western Australian Museum in Perth (WAM) to access their *Pegasus* collections.

### Specimens Examined

A total of 135 specimens of the new species were used for the description. Most of these specimens were collected by scientific trawling in the Torres Strait and Great Barrier Reef between 2003 and 2005 [27,28]. Of these, 104 (including the holotype) are deposited at the Australian National Fish Collection in Australia (CSIRO), 17 specimens at the Australian Museum (AMS), 4 specimens at the Museum and Art Gallery of the Northern Territory (NTM), 6 specimens at the Queensland Museum (QM), 2 at the Western Australian Museum (WAM), one at the Smithsonian Institution in the USA (USNM), and one at the Naturhistoriska Riksmuseet in Sweden (NRM). Muscle tissue samples were taken from a subsample of these specimens and were stored frozen. Whole retained specimens were fixed in a 10% formalin solution and later transferred into 70% ethanol for long-term preservation.

Comparative material of *Pegasus lancifer* and *P*. *volitans* examined in this study are presented in S1 Text.

### Morphology

As mentioned by [14], the unusual body profile of pegasids requires a modification of standard measurements. The measurement methodology adopted for pegasids in this paper is illustrated in S1 Fig and detailed in S1 Table, and the configuration of the external plates is illustrated in S2 Fig. Standard length, following [14], was taken from the anteriormost extent of the maxillae (with mouth closed) to the posteriormost margin of the hypural plate. Since most collections record standard length for pegasids as from the tip of the rostrum to the posteriormost margin of the hypural plate, the material examined sections include this measurement, defined here as precaudal length (PCL). The length, width and height of all 12 tail rings were measured, but since there were no significant differences in these measurements for tail rings I–IX between the new species and *Pegasus volitans*, only the measurements for tail rings X–XII are presented in Table 1.

**Table 1:** Morphometric data for *Pegasus tetrabelos* and *P*. *volitans*. Proportional measurements of the holotype (CSIRO H 6553–03) and 13 paratypes of *P*. *tetrabelos*, and 10 specimens of *P*. *volitans* from Queensland as percentages of standard length.

	*Pegasus tetrabelos* sp. nov.				*Pegasus volitans*		
	Holotype	Paratypes (n = 13)			(n = 10)		
Measurement		Min.	Max.	Mean	Min.	Max.	Mean
SL—standard length (mm)	91.1	78.8	97.6	87.2	82.2	114.6	98.1
PCL—precaudal length	121.0	118.1	125.4	121.2	112.8	125.4	120.4
CaL—carapace length	34.6	31.3	36.8	34.3	31.4	36.3	33.4
PP2 –prepelvic length	54.2	50.1	55.6	52.8	44.3	55.4	51.9
PAN—preanal length	61.3	56.9	63.6	59.7	51.2	62.0	58.5
TaL—tail length	60.5	59.3	65.2	62.2	59.2	65.3	62.4
PPW—prepectoral width	20.7	18.4	21.5	20.1	17.3	19.2	18.1
IPW—interpectoral width	17.3	14.1	17.6	15.8	12.7	15.5	14.1
CaW—carapace width	16.5	13.5	18.1	16.1	12.8	15.5	14.4
BD—body depth	8.3	6.8	9.5	7.8	6.3	8.9	7.7
RoL—rostrum length	25.4	22.5	29.5	25.1	21.3	29.3	25.7
RoW—rostrum width	5.4	5.0	6.9	5.9	4.8	6.8	5.6
RoW-tip—rostrum width at tip	3.5	2.0	3.6	2.7	2.2	4.4	3.0
INO—interorbital width	5.0	4.5	5.5	5.0	4.5	5.3	4.9
HDW—head width	16.1	14.3	16.7	15.7	13.7	16.2	14.8
HDH—head height	7.2	6.3	7.4	7.0	6.4	7.7	7.1
HDW-eye—head width at posterior orbit	13.2	12.0	13.9	12.9	10.6	12.2	11.7
OrL—orbit length	6.1	5.5	7.2	6.2	5.5	6.0	5.7
OrW—orbit height	4.6	4.1	5.5	4.6	4.4	5.2	4.9
P1L-5th—length of 5th pectoral ray	23.0	20.8	26.7	24.5	21.7	29.1	26.0
D1B —dorsal-fin base length	7.9	7.7	9.5	8.4	8.5	10.3	9.2
ANB—anal-fin base length	7.6	6.1	8.3	7.2	6.6	9.5	8.2
CFR- upper—length of upper caudal ray	12.0	11.6	13.8	12.9	11.7	13.6	12.7
TaRL-X—length of X tail ring	8.4	5.4	10.2	8.0	7.5	10.6	9.0
TaRW-X—width of X tail ring	4.5	4.1	5.0	4.5	3.4	4.0	3.7
TaRH-X—height of X tail ring	1.4	1.0	1.8	1.4	1.1	1.6	1.4
TaRL-XI—length of XI tail ring	6.1	5.4	7.4	6.4	6.3	9.5	7.7
TaRW-XI—width of XI tail ring	3.4	3.1	4.4	3.7	2.5	3.4	3.1
TaRH-XI—height of XI tail ring	1.3	0.8	1.3	1.0	0.8	1.1	0.9
TaRL-XII—length of XII tail ring	2.6	2.2	3.6	3.0	2.3	4.7	3.8
TaRW-XII—width of XII tail ring	2.6	2.3	3.2	2.7	2.8	3.5	3.2
TaRW-XII—height of XII tail ring	1.8	1.3	2.0	1.7	1.0	1.4	1.1
SpL—length of XII tail ring spine	1.9	2.0	3.0	2.5	4.1	5.5	4.9

Full morphometric data were collected for the holotype and 13 paratypes of the new species (Table 1). The paratypes measured were: CSIRO H 6543–04, CSIRO H 6543–05, CSIRO H 7090–04, CSIRO H 7491–03, CSIRO H 7665–01, CSIRO H 7667–02, CSIRO H 7667–03, CSIRO H 7673–02 (3 specimens), CSIRO H 7676–02 (2 specimens), and CSIRO H 7678–01. For comparison, 10 specimens of *Pegasus volitans* from the same geographic area (Torres Strait and Great Barrier Reef) were measured in full. These specimens were: CSIRO H 6510–03, CSIRO H 6553–04, CSIRO H 6649–02, CSIRO H 6738–04, CSIRO H 6901–05, CSIRO H 6901–06, CSIRO H 6903–03, CSIRO H 7665–02, CSIRO H 7669–01, and USNM 434843 (see S1 Text).

To determine sex by gonad examination, 33 frozen specimens were thawed and dissected. Once several females and males could be accurately identified, a subset of morphological characters previously found to be useful in distinguishing between sexes of *Pegasus* species were examined and tested for consistency (see [14,29]. Resulting sexual dimorphic characters were used to determine the sex of all but one of the measured types listed above.

In the comparison with other species, the data for *P*. *latenarius* and *P*. *lancifer* are taken from [14], except for the information on ventral preopercular notches on *P*. *lancifer* which was made based on material examined in this study. Data for the new species and *P*. *volitans* are from the present study.

### Meristics

The strongly calcified dermal plates of pegasids present a significant challenge to obtaining accurate vertebral counts, especially in the precaudal region. To negate the interference of this external body armour, a selection of paratypes which were dissected for sex determination were radiographed. The plastron and body cavity organs were removed from these specimens, enabling easier recognition of vertebral column elements. Vertebral count methodology and terminology follows [14,30]; counts are presented as abdominal, caudal and total centra.

Counts of vertebrae were made from radiographs of the following specimens of the new species: CSIRO H 6520–02 (1 of 3 specimens), CSIRO H 6914–04, CSIRO H 7675–02 (1 of 4 specimens), CSIRO H 7684–01, and CSIRO H 7687–01. For comparison, radiographs were also taken from the following specimens of *Pegasus volitans*: CSIRO H 6507–04, CSIRO H 6903–04, CSIRO H 7674–02, CSIRO H 7692–01 (2 specimens), and CSIRO H 7693–01.

Fin ray counts were taken from all measured specimens of the new species and *Pegasus volitans* (see Morphology section).

### DNA barcoding

Here we used two mtDNA genes alongside the morphological and meristic data for cryptic species determination in *P*. *volitans*. The *16S* gene is relatively widely used for species identifications and phylogenetic analyses, while *COI*, our preferred ‘barcode’ gene of choice for Australian fishes [4,5] is also analysed. There are few publically available sequences for *Pegasus*; however GenBank contains several examples of full mtDNA genome information for *P*. *volitans* and *E*. *draconis* while there are there are only two publically available records of COI sequences for *P*. *volitans* on BOLD. Specimens from which tissues samples for DNA barcoding were taken are in S2 Table. The specific methodology, DNA extraction protocols and sequencing processes for the two genes are provided in S2 Text.

### Nomenclatural Acts

The electronic edition of this article conforms to the requirements of the amended International Code of Zoological Nomenclature, and hence the new names contained herein are available under that Code from the electronic edition of this article. This published work and the nomenclatural acts it contains have been registered in ZooBank, the online registration system for the ICZN. The ZooBank LSIDs (Life Science Identifiers) can be resolved and the associated information viewed through any standard web browser by appending the LSID to the prefix “http://zoobank.org/”. The LSID for this publication is: urn:lsid:zoobank.org:pub:5CEA3535-4A26-4447-B42E-8ED17B368E9E. The electronic edition of this work was published in a journal with an ISSN, and has been archived and is available from the following digital repositories: PubMed Central and LOCKSS.

## Results

### Diagnosis and Description

*Pegasus tetrabelos* Osterhage, Pogonoski, Appleyard and White sp. nov. urn:lsid:zoobank.org:act:359804F0-30D0-4ECC-B355-D60D45556018.

(Figs 2, 3a, 4a, 5a, 6a, 7a, 8–10; Table 1)

**Fig 2:**
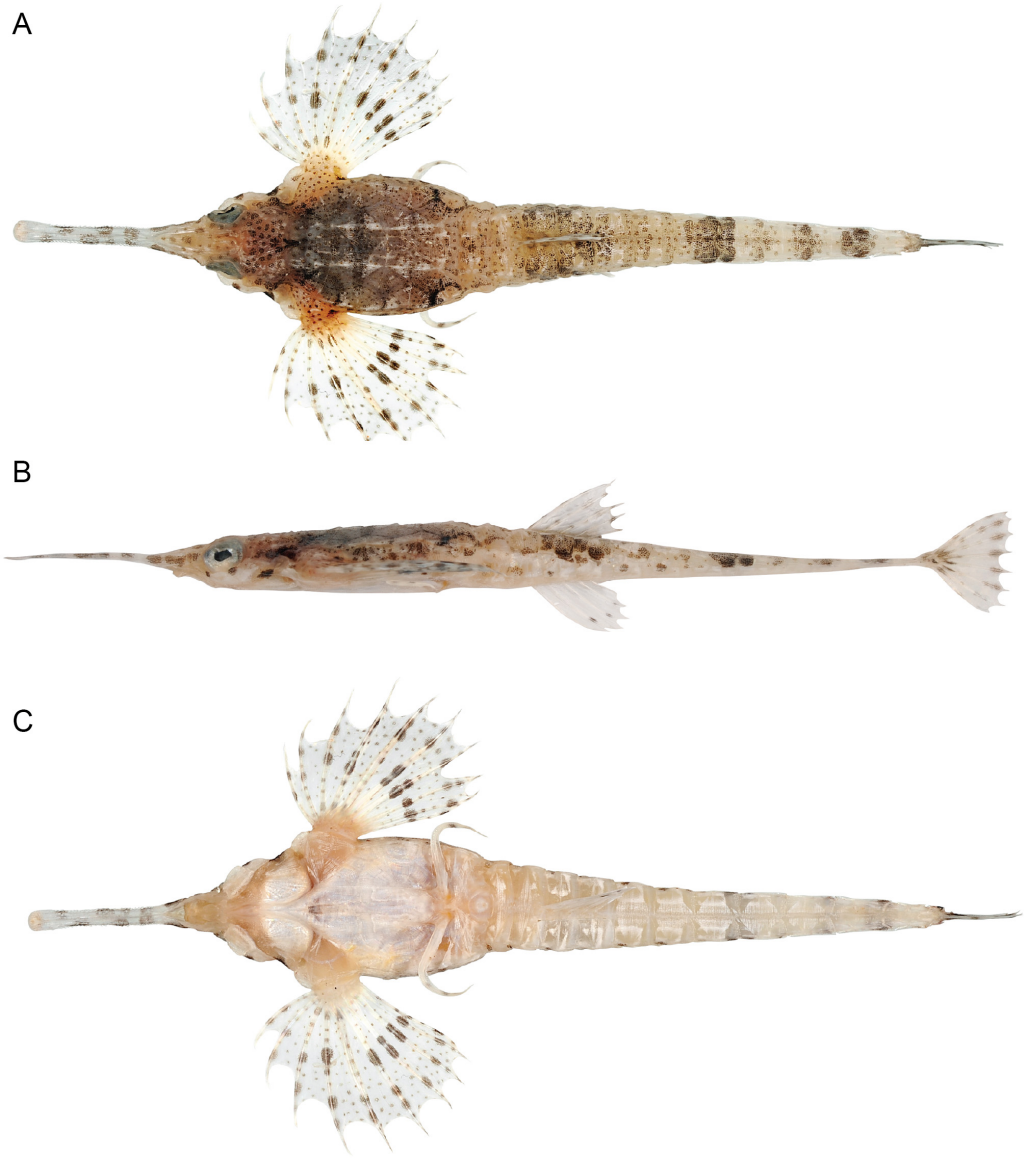
Holotype of *Pegasus tetrabelos* (CSIRO H 6553–03, 110 mm PCL). (A) dorsal; (B) lateral; and (C) ventral views.

**Fig 3:**
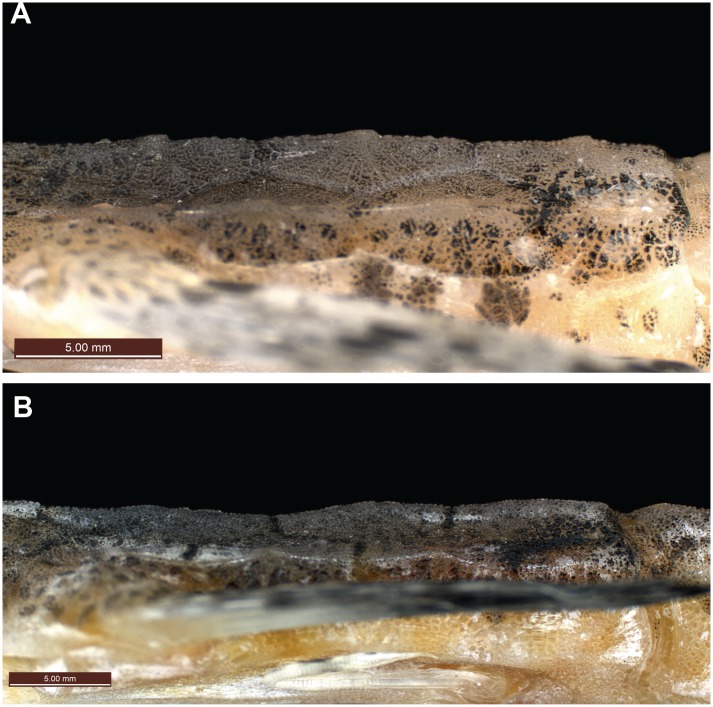
Lateral view of dorsal ridge. (A) *Pegasus tetrabelos* (CSIRO H 7665–01); (B) *Pegasus volitans* (CSIRO H 6649–02).

**Fig 4:**
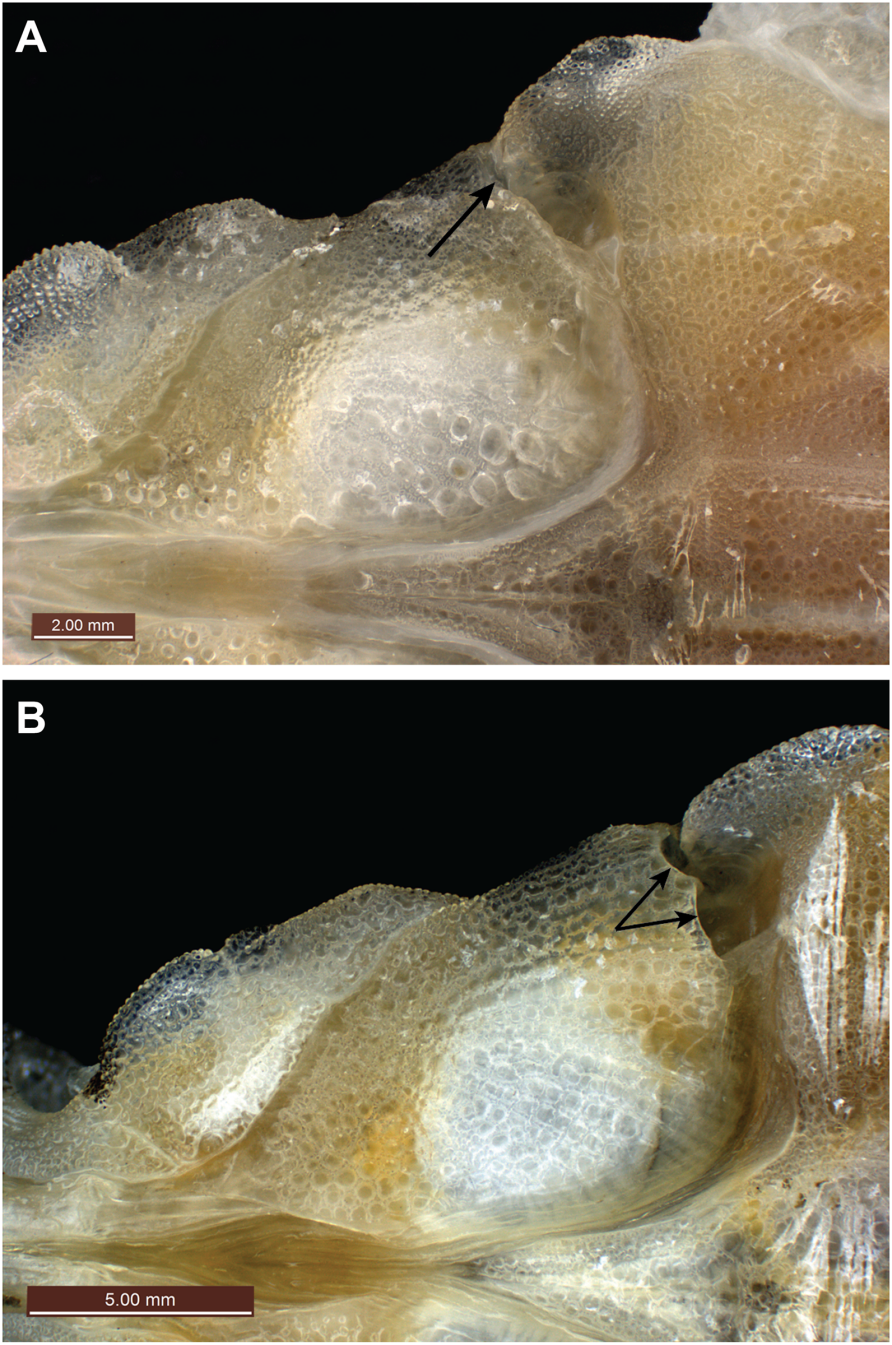
Ventral view of preopercular plate. (A) *Pegasus tetrabelos* (CSIRO H 7665–01), arrow indicates single ventral preopercular notch; (B) *Pegasus volitans* (CSIRO H 6649–02), arrows indicate double ventral preopercular notches.

**Fig 5:**
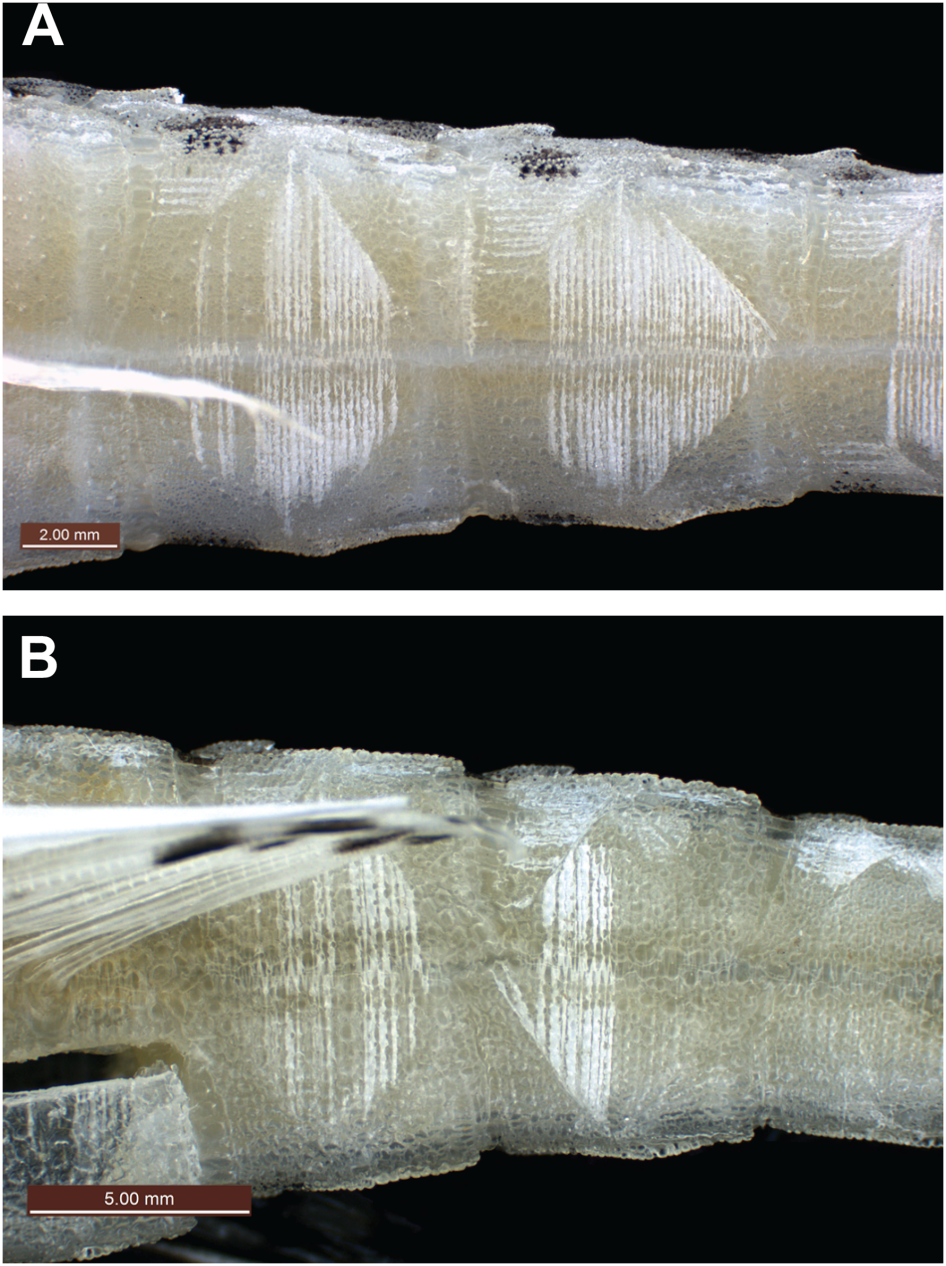
Ventral view of tail from mid-tail ring IV to mid tail ring VII. Caudolateral plate keels at intersects of tail rings. (A) *Pegasus tetrabelos* (CSIRO H 7665–01); (B) *Pegasus volitans* (CSIRO H 6649–02).

**Fig 6:**
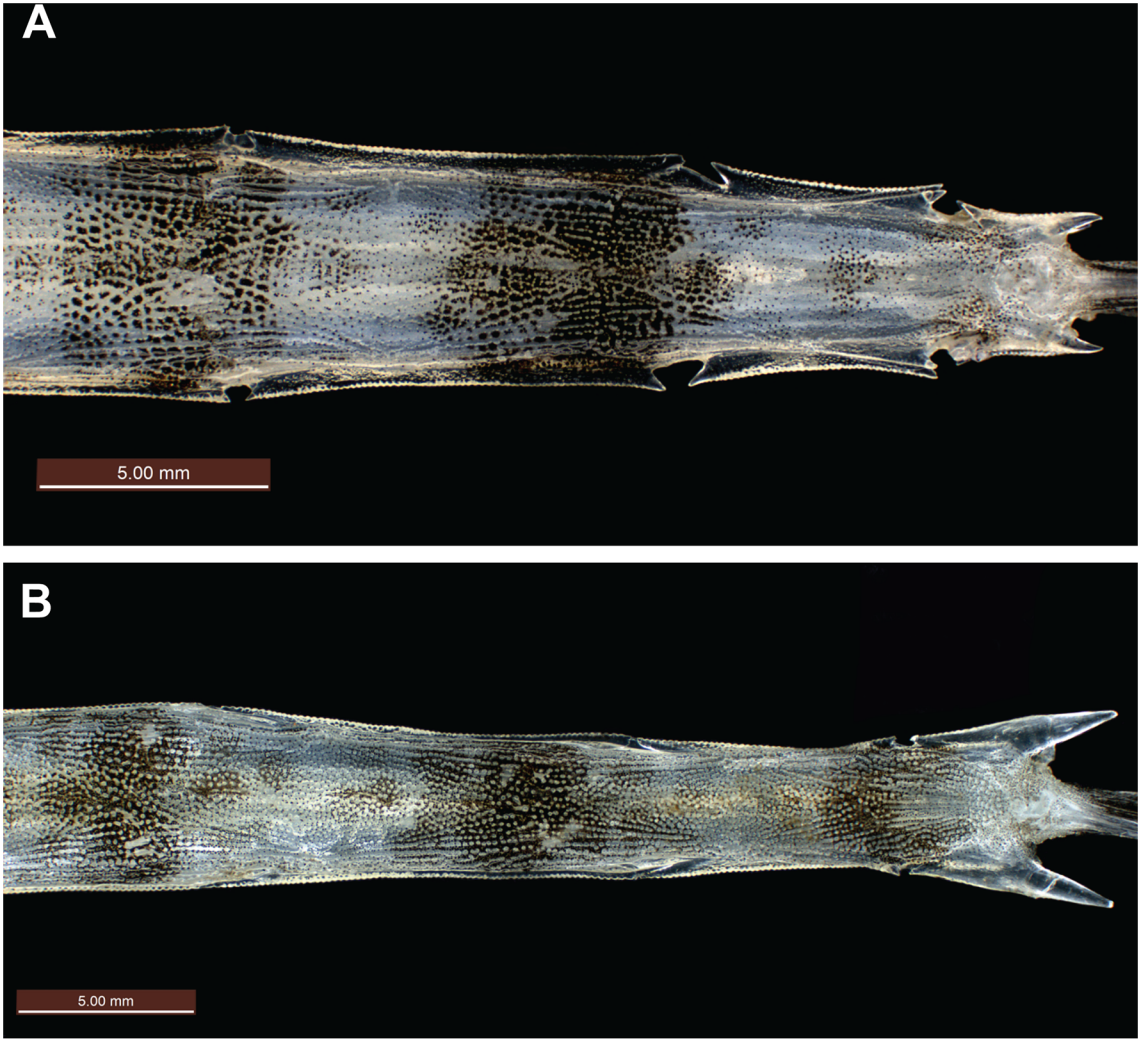
Dorsal view of posterior tail from mid-tail ring X to tail ring XII. (A) *Pegasus tetrabelos* (CSIRO H 7665–01); (B) *Pegasus volitans* (CSIRO H 6649–02).

**Fig 7:**
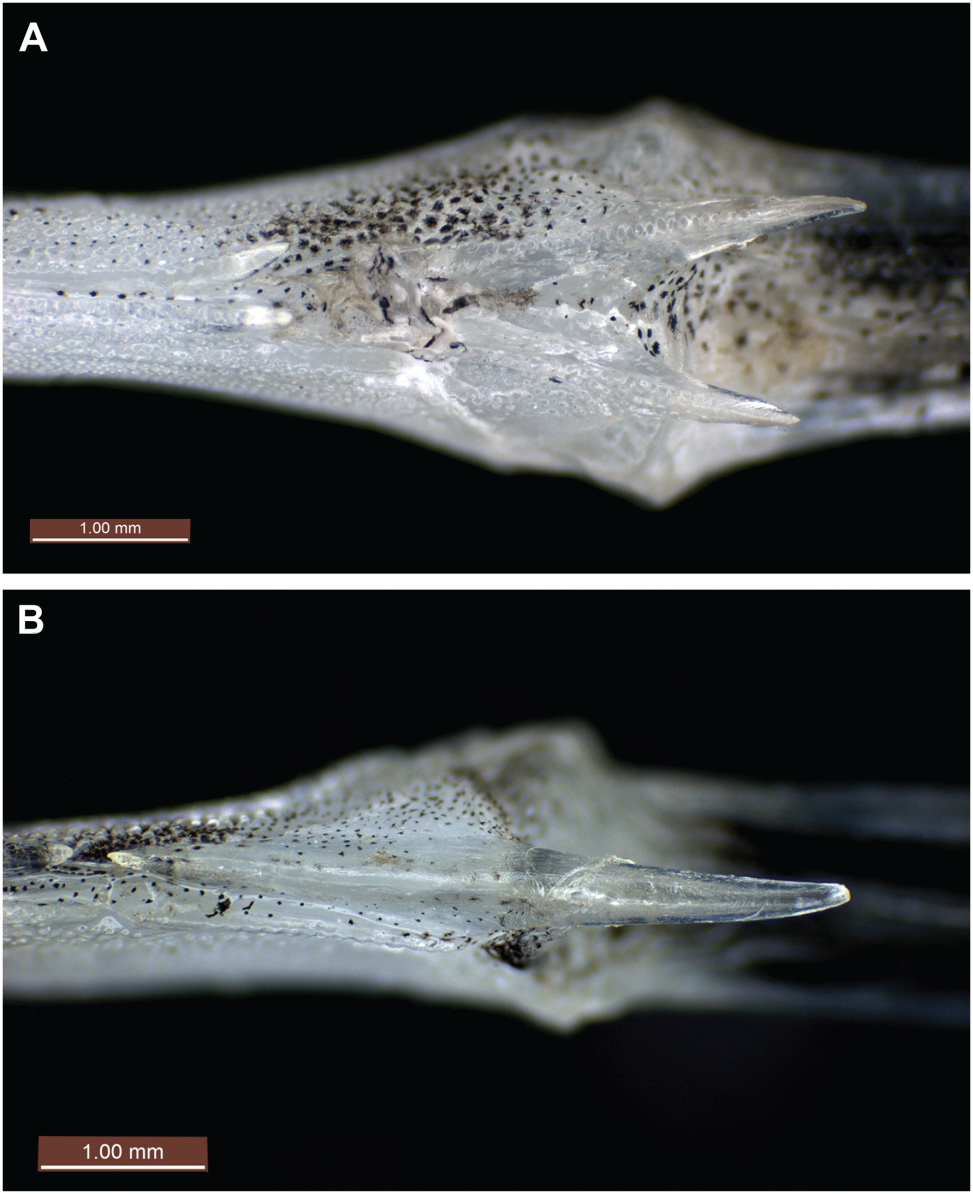
Lateral view of tail ring XII. (A) *Pegasus tetrabelos* (CSIRO H 7665–01) showing terminodorsal-lateral and terminoventral-lateral plates each with an anteriorly and posteriorly directed spine; (B) *Pegasus volitans* (CSIRO H 6649–02) showing terminal-lateral plate with an anteriorly and posteriorly directed spine.

**Fig 8:**
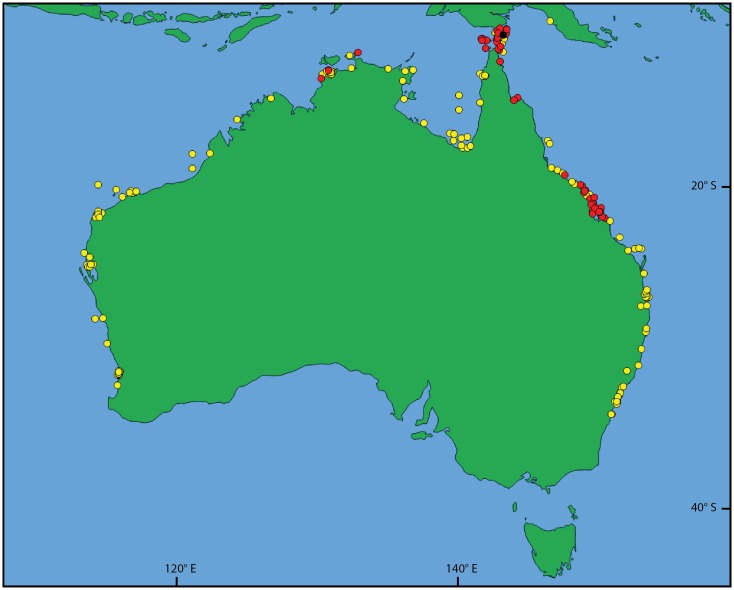
Map showing the collection locations of the material examined. *Pegasus volitans* specimens indicated by yellow dots and *Pegasus tetrabelos* specimens indicated by black (holotype) and red (paratypes) dots. Map generated in QGIS using Natural Earth 1:10,000,000 data (www.naturalearthdata.com).

**Fig 9:**
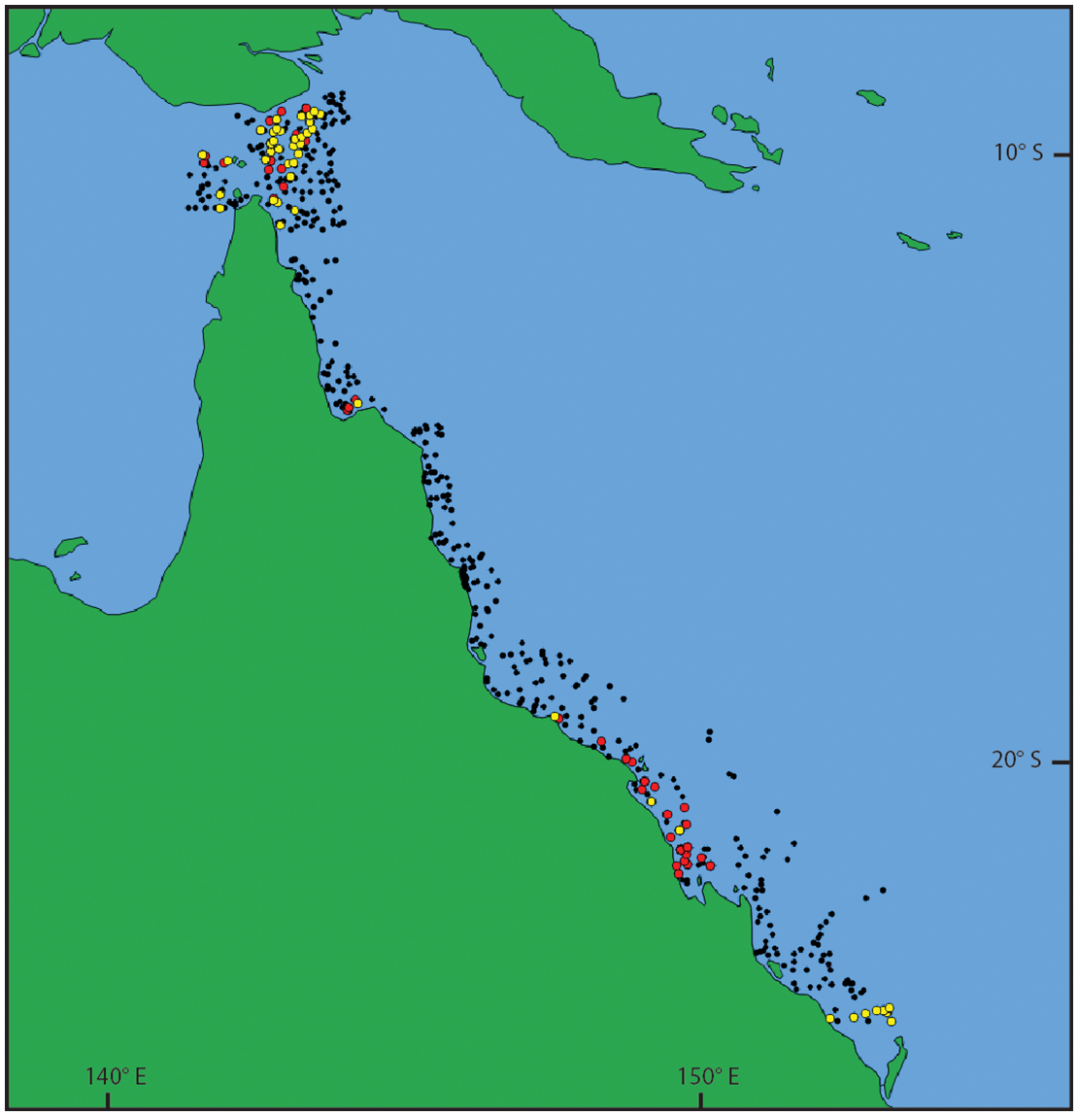
Trawl sites from the Torres Strait and Great Barrier Reef surveys. Sites where *Pegasus tetrabelos* (red dots) and *Pegasus volitans* (yellow dots) were recorded; black dots refer to those sites where *Pegasus* specimens were not captured. Map generated in QGIS using Natural Earth 1:10,000,000 data (www.naturalearthdata.com).

**Fig 10:**
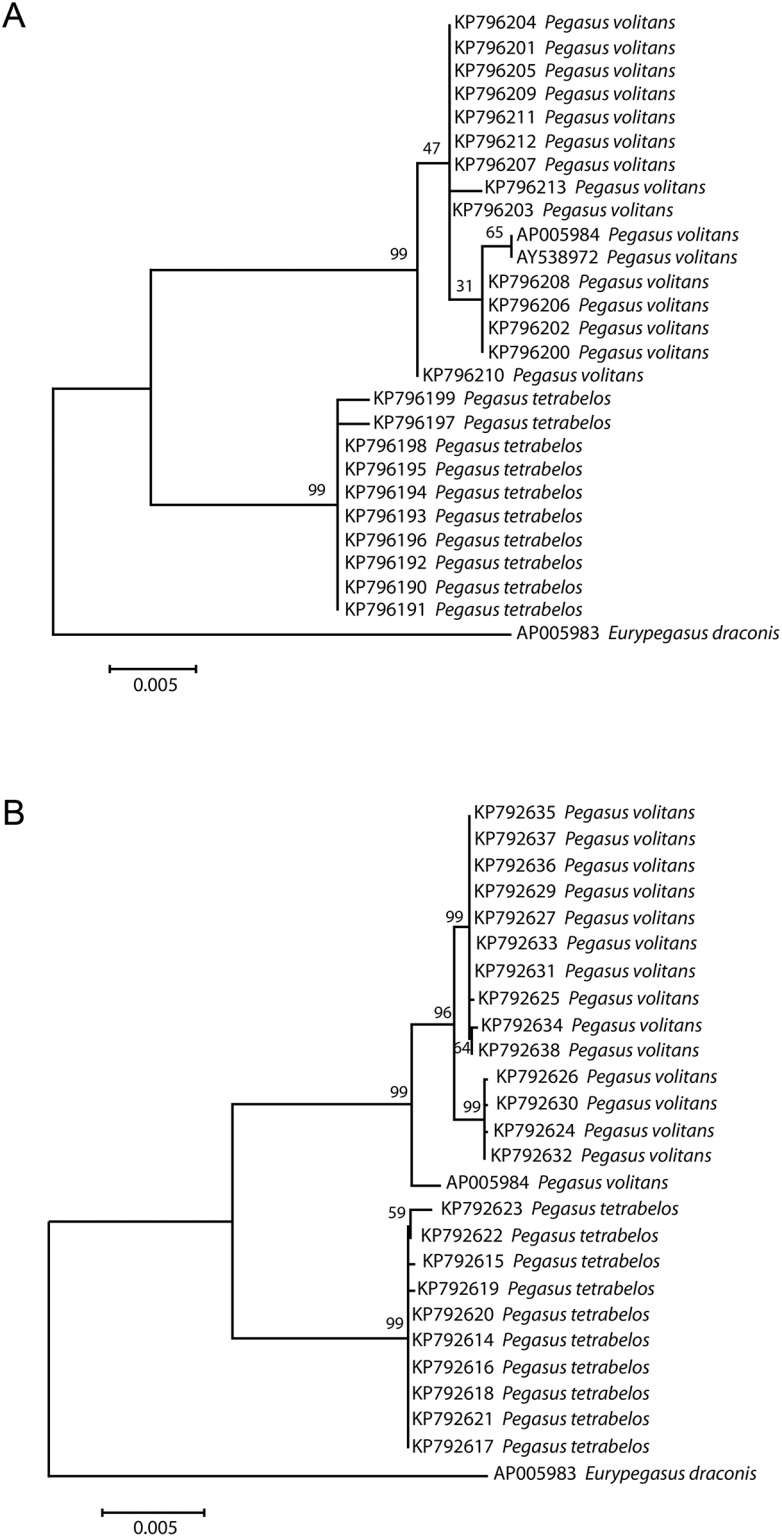
Molecular species identification of *Pegasus* species using Genetic treeML trees. (A) sequences from the *16S* gene; (B) sequences from the *COI* gene. Trees are based on the K2 evolutionary distance model and are shown here with mined *Pegasus* and *Eurypegasus* sequences from GenBank. The trees are shown here with an *E*. *draconis* outgroup. Bootstrap support values (following 1000 replicates) are shown above the nodes.

#### Material

Holotype. CSIRO H 6553–03, female 110 mm PCL, northeast of Dungeness Island, Torres Strait, Australia, 09°46’9” S, 143°09’19” E, 19 m depth, 24 Jan 2004.

Paratypes. Northern Territory, Australia: NTM S. 10604–008, 32 mm PCL, near North Oxley Island, Oxley Islands, 11° S, 132°49’1” E, 10 m depth, 19 Oct 1982; NTM S. 13283–003, 115 mm PCL, northeast of Charles Point, Beagle Gulf, 12°15’ S, 130°40’58” E, 26 m depth, 2 Sep 1992; NTM S. 13284–003 (2 specimens), 99–119 mm PCL, northeast of Charles Point, Beagle Gulf, 12°16’58” S, 130°40’ E, 24 m depth, 2 Sep 1992; WAM P. 29139–002, 109+ mm PCL (rostrum tip damaged), ~84 km southwest of Darwin, 12°50’ S, 130°10’ E, 34–45 m depth, 10 Sep 1965.

Queensland, Australia: AMS IA. 6761 (2 specimens), 57–60 mm PCL, Lindeman Island, 20°27’ S, 149°02’ E, 1936; AMS I. 20771–097 (6 specimens), 109–120 mm PCL, 1–9 mile east of Captain Billy Creek, Cape York, 11°37’ S, 142°56’ E, 16–18 m depth, 18 Feb 1979; AMS I. 34375–001 (9 specimens), 75–92 mm PCL, 5 km southwest of Leicester Island, Shoalwater Bay, 22°18’54” S, 150°22’44” E, 16 m depth, 24 Oct 1993; CSIRO H 6520–02 (2 males, 1 female), 73–102 mm PCL, north of Broad Sound, 21°47’48” S, 149°32’12” E, 10 m depth, 13 Nov 2005; CSIRO H 6543–03, 107 mm PCL, CSIRO H 6543–04, male 109 mm PCL, CSIRO H 6543–05, male 97 mm PCL, east of Banks Island, Torres Strait, 10°07’46” S, 142°45’51” E, 19 m depth, 12 Jan 2004; CSIRO H 6547–02 (1 male, 1 female), 100–123 mm PCL, east of Newcastle Bay, 10°45’27” S, 142°47’21” E, 26 m depth, 25 Sep 2004; CSIRO H 6555–02, male 102 mm PCL, northeast of Mackay, 21°01’34” S, 149°21’26” E, 25 m depth, 29 Apr 2004; CSIRO H 6667–03, 114 mm PCL, north of Dungeness Island, Torres Strait, 9°26’57” S, 142°51’12” E, 12 m depth, 23 Jan 2004; CSIRO H 6688–02, male 110 mm PCL, west of Mulgrave Island, Torres Strait, 10°10’33” S, 141°58’39” E, 12 m depth, 17 Jan 2004; CSIRO H 6738–06 (3 specimens), 114–120 mm PCL, CSIRO H 6738–07, female 110 mm PCL, southeast of Mackay, 21°17’58” S, 149°34’11” E, 21 m depth, 29 Apr 2004; CSIRO H 6793–02, male 115 mm PCL, east of Banks Island, Torres Strait, 10°16’41” S, 142°42’31” E, 19 m depth, 13 Jan 2004; CSIRO H 6795–03 (2 specimens), 101–106 mm PCL, west of Prince of Wales Island, Torres Strait, 10°41’38” S, 141°53’36” E, 14 m depth, 18 Jan 2004; CSIRO H 6912–06, female 112 mm PCL, east of Saibai Island, Torres Strait, 9°18’34” S, 142°55’04” E, 11 m depth, 23 Jan 2004; CSIRO H 6914–04, female 114 mm PCL, west of Mulgrave Island, Torres Strait, 10°02’43” S, 141°36’32” E, 16 m depth, 16 Jan 2004; CSIRO H 6918–04 (2 specimens), 91–92 mm PCL, east of Bowling Green Bay, 19°26’48” S, 147°34’ E, 16 m depth, 2 Dec 2003; CSIRO H 7090–02 (15 specimens), 88–121 mm PCL, CSIRO H 7090–03, 103 mm PCL, CSIRO H 7090–04, female 107 mm PCL, CSIRO H 7090–05 (dry), ~109 mm PCL, CSIRO H 7090–06, 88 mm PCL, north of Broad Sound, 21°37’51” S, 149°36’03” E, 15 m depth, 28 Apr 2004; CSIRO H 7491–03, male 109 mm PCL, west of Mulgrave Island, Torres Strait, 10°02’55” S, 141°39’37” E, 14 m depth, 16 Jan 2004; CSIRO H 7502–03 (2 females, rostra damaged), 86+ and 95+ mm PCL, south of Whitsunday Island, 20°29’32” S, 148°59’21” E, 31 m depth, 5 Dec 2003; CSIRO H 7660–03 (5 specimens), 102–115 mm PCL, east of Northumberland Islands, 21°37’22” S, 150°07’47” E, 34 m depth, 28 Nov 2005; CSIRO H 7665–01, 111 mm PCL, CSIRO H 7665–03, female 110+ mm PCL (rostrum tip damaged), north of Dalrymple Island, Torres Strait, 9°21’55” S, 143°24’18” E, 20 m depth, 25 Jan 2004; CSIRO H 7666–01 (3 specimens), 105–120 mm PCL, west of Hook Island, 20°09’46” S, 148°46’43” E, 27 m depth, 4 Dec 2003; CSIRO H 7667–01, 74 mm PCL, CSIRO H 7667–02, female 103 mm PCL, CSIRO H 7667–03, 102 mm PCL, south of Saibai Island, Torres Strait, 9°28’6” S, 142°43’35” E, 8 m depth, 23 Jan 2004; CSIRO H 7668–01 (3 specimens), 64–74 mm PCL, north of Broad Sound, 21°52’58” S, 149°30’56” E, 10 m depth, 28 Apr 2004; CSIRO H 7673–02 (2 females; 1 male), 102–116 mm PCL, east of Banks Island, Torres Strait, 10°06’44” S, 142°39’38” E, 17 m depth, 12 Jan 2004; CSIRO H 7674–01 (2 specimens), 89–99 mm PCL, east of Flinders Group, 14°10’48” S, 144°11’38” E, 15 m depth, 21 Sep 2004; CSIRO H 7675–01 (2 specimens), both 71 mm PCL, CSIRO H 7675–02 (1 male, 3 females), 66–73 mm PCL, Broad Sound, 22°01’ S, 149°32’56” E, 11 m depth, 28 Apr 2004; CSIRO H 7676–01 (3 specimens), 98–107 mm PCL, CSIRO H 7676–02 (3 females), 98–108 mm PCL, CSIRO H 7676–03, female 102 mm PCL, west of Northumberland Islands, 21°35’36” S, 149°42’15” E, 21 m depth, 28 Apr 2004; CSIRO H 7677–01, 108 mm PCL, west of Double Cone Island, 20°06’33” S, 148°41’10” E, 30 m depth, 4 Dec 2003; CSIRO H 7678–01, male 94 mm PCL, CSIRO H 7678–02 (2 female, 1 male), 90–94 mm PCL, northeast of Broad Sound, 21°53’35” S, 150°04’48” E, 22 m depth, 9 Dec 2003; CSIRO H 7679–01 (10 specimens), 86–105 mm PCL, north of Broad Sound, 21°44’17” S, 149°36’11” E, 14 m depth, 12 Nov 2005; CSIRO H 7683–01, female 108 mm PCL, north of Ince Bay, 21°24’57” S, 149°25’36” E, 10 m depth, 29 Apr 2004; CSIRO H 7684–01, female 108+ mm PCL (rostrum tip damaged), south of Scawfell Island, Cumberland Island group, 20°55’32” S, 149°38’22” E, 40 m depth, 29 Apr 2004; CSIRO H 7685–01 (2 females), 110–115 mm PCL, west of Curlew Island, Northumberland Island Group, 21°41’18” S, 149°41’38” E, 18 m depth, 28 Apr 2004; CSIRO H 7686–01, male 114 mm PCL, northeast of Newcastle Bay, Torres Strait, 10°34’23” S, 142°58’22” E, 22 m depth, 9 Jan 2004; CSIRO H 7687–01, female 115 mm PCL, west of Mulgrave Island, Torres Strait, 10°09’48” S, 141°38’36” E, 15 m depth, 16 Jan 2004; CSIRO H 7688–01, male 118 mm PCL, northwest of Long Island, Torres Strait, 9°58’51” S, 142°45’53” E, 18 m depth, 20 Jan 2004; CSIRO H 7689–01, male 111 mm PCL, northeast of Dungeness Island, Torres Strait, 9°39’29” S, 142°47’36” E, 10 m depth, 23 Jan 2004; CSIRO H 7690–01, female 109 mm PCL, northeast of Broad Sound, 21°54’52” S, 150°01’58” E, 21 m depth, 13 Nov 2005; CSIRO H 7691–01, male 91 mm PCL, Princess Charlotte Bay, 14°19’19” S, 143°54’44” E, 8 m depth, 3 Mar 2006; NRM 66669, 108 mm PCL, north of Broad Sound, 21°37’51” S, 149°36’03” E, 15 m depth, 28 Apr 2004; QM I. 36186 (3 specimens), 91–103 mm PCL, northwest of Osborne Island, 22°13’ S, 150°13’ E, 21 m depth, 10 May 2004; QM I. 36040 (3 specimens), 104–110 mm PCL, east of Repulse Islands, 20°36’5” S, 148°55’59” E, 24 m depth, 30 Nov 2003; WAM P. 34325–001, 116 mm PCL, east of Newcastle Bay, Torres Strait, 10°50’49” S, 142°52’43” E, 25 m depth, 25 Sep 2004; USNM 434842, 113 mm PCL, south of Dalrymple Island, Torres Strait, 9°41’05” S, 143°21’41” E, 25 m depth, 24 Jan 2004.

Other specimens. AMS IA. 6803, 11 mm PCL, Lindeman Island, Queensland, Australia, 20°27’ S, 149°02’ E, 1936; NTM S. 13721–009, 12 mm PCL, south of Mitchell Creek, Darwin Harbour, 12°30’46” S, 130°56’23” E, 7 m depth, 16 Jul 1993.

#### Diagnosis

Tail rings 12, anteriormost 9 mobile, articulating laterally, remaining 3 fused together, attenuated and dorsoventrally flattened; terminodorsal-lateral (tdl) and terminoventral-lateral (tvl) plates each with an anteriorly and posteriorly directed spine; terminal-lateral plates (tl) absent; interpectoral plate (ip) present; single ventral preopercular notch present; rostrum spatulate; carapace with three small posteriorly directed tubercles along each dorsal ridge, one at the centre of each dorsal plate; scales not present on orbit; pectoral fin composed of 9–10 (usually 10) soft rays, 5^th^ ray stouter than other rays; abdominal centra 7, caudal centra 14, total centra 21; tail with 4 dark saddles, no dark saddle on tail ring XI.

#### Description

Body depressed, carapace depth 8.3 in holotype (6.8–9.5 in paratypes)% SL, carapace depth generally constant; interpectoral width 17.3 (14.1–17.6)% SL; carapace width 16.5 (13.5–18.1)% SL; body width gradually tapering from mid-trunk to tail. Rostrum long, length 25.4 (22.5–29.5)% SL, tapering from anterior orbit to anterior subrostral chamber then about constant throughout its length; slightly club-shaped anteriorly, tip rounded (straight to club-shaped, tip rounded to truncate in paratypes), width at tip 3.5 (2.0–3.6)% SL; rostral spines present from rostrum tip to anterior subrostral chamber (sometimes covered with skin), 35 each side in holotype (often damaged in paratypes: 33–41 on left side [n = 7], 30–38 [n = 8] on right side). Orbit subcircular; length 6.1 (5.5–7.2)% SL, height 4.6 (4.1–5.5)% SL; interorbital width 5.0 (4.5–5.5)% SL, narrowest at posterior pupil; shallow interorbital depression; suborbital spines absent; parietals and epioccipitals with small tubercles. Carapace length 34.6 (31.3–36.8)% SL; low, paired dorsal ridges starting posterior to orbit and bisecting dorsal plates, parallel but widening slightly at carapace base, broadening onto anterior tail rings to form dorsolateral margin of tail; 3 small posteriorly directed tubercles on each dorsal ridge, one at the centre of each dorsal plate (Fig 3A); weak transverse ridges interconnecting dorsal plate tubercles; shallow depressions formed by lattice of dorsal ridges and transverse ridges. Plates vl_3–5_ fold over plates dl_2–4_, forming a grooved lateral carapace ridge between pectoral fin insertion and lateral junction of tail ring I; dorsolateral carapace plates each with a weak posteriorly directed tubercle; ventrolateral plates II to V with weak tubercles on lateral edges (weak to distinct in paratypes). A single ventral preopercular notch present where anterior point of vl_1_ slots into a single notch at outer junction of vl_1_ and preopercular plate (Fig 4A). Body, rostrum, head, and tail covered in tiny denticles; denticles in longitudinal rows on rostrum; scales absent.

Tail with 11 paired caudodorsal plates (cd_1–11_), 11 paired caudoventral plates (cv_1–11_), an unpaired termino-dorsal plate (td), an unpaired termino-ventral plate (tv), and terminodorsal-lateral (tdl) and terminoventral-lateral (tvl) plates each with an anteriorly and posteriorly directed spine; terminal-lateral plates (tl) absent; 6 paired caudolateral plates (cl_1–6_), cl_1_ is on posterolateral side of tail ring I and does not overlap tail ring II; cl_2_ overlaps tail rings II–III, cl_3_ overlaps tail rings III to IV, cl_4_ overlaps tail rings IV to V, cl_5_ overlaps tail rings V to VI, and cl_6_ overlaps tail ring VI to VII; lateral plates are scutella-like anteriorly and keel-like posteriorly; keel on cl_1_ is largest, approximately ¾ visible tail ring length, elevated compared to remaining keels and broadly rounded; keels on remaining caudolateral plates posteriorly directed (Fig 5A) and roughly diamond-shaped. Tail rings I–VIII rectangular in cross-section, with dorsal, ventral, and lateral faces joined at dorsolateral and ventrolateral margins; tail rings sequentially tapering to form a dorsoventrally flattened posterior section. Small tubercle on centre of each caudodorsal plate, most prominent anteriorly, and decreasing in size and barely visible beyond tail ring IX. Tail rings I–VIII with weak tubercles/keels at ventrolateral margin. Tail rings VIII–XII with translucent, lateral ridges that extend length of tail ring; each lateral ridge with fine denticulations along its length except where ending in a spine tip; tail ring IX ridge terminating in small single posteriorly-directed spine (spines sometimes encased within skin); tail ring X and XI ridges starting and ending with small single anterior and posterior spines; tail ring XI also with superior ridge starting mid-tail ring and terminating in an additional posterior spine; terminodorsal-lateral (tdl) and terminoventral-lateral (tvl) plates each with an anteriorly and posteriorly directed spine, plates diverge posteriorly with distance between posterior spines twice the distance between anterior spines (Fig 7A).

Pectoral fin with 10 soft rays (9 on both sides of paratype CSIRO H 6543–04), 5^th^ ray stouter than other rays. Pelvic fin with 1 spine and 2 rays; 2^nd^ ray reduced to a single element, very small in size and connected by thin membrane to 1^st^ ray. Dorsal fin with 5 rays; anal fin with 5 rays; length of dorsal and anal fin rays not equal, anterior ray longest, posterior ray shortest. Caudal fin with 8 rays, truncate to slightly convex.

#### Colour

Holotype (female, Fig 2): Prior to preservation (post-thawing, specimen frozen for 10 years). Dorsal and lateral body surfaces cream to medium brown, overlaid with dark brown to black spots, blotches and saddles. Trunk medium brown with small dark brown to black spots; a dark brown to black transverse bar across plate dl_2_; dorsal ridges cream; plate dl_4_ with black border, thickest anteriorly at margin with plate dl_3_. Head cream to reddish-brown with pale brown to black spots; a triangular pattern of dark brown spots between posterior orbits and anterior junction of plates dl_1_, with a small, dark V-shaped marking posteriorly; three prominent dark brown to black spots about half pupil width, first below anterior eye (only visible laterally), second below posterior pupil, and third anterior of 3^rd^ circumorbital (second and third spots visible dorsally, laterally and ventrally). Rostrum translucent, in dorsal view from posterior to anterior: small dark brown spots between orbit and above subrostral chamber, a pair of large pupil-sized spots wrapping around lateral edges in line with anterior subrostral chamber, followed by a single dark brown bar and 5 roughly pupil-sized dark brown spots that alternate along the rostrum edges, ending just before the rostral tip; in lateral view: 3 horizontal blotches between anterior orbit and level of anterior subrostral chamber; in ventral view: no additional pigmentation, but dorsal and lateral markings visible through translucent section of rostrum. Pectoral-fin base cream to pale-brown with small reddish-brown spots. Pectoral fins translucent with small dark brown spots on fin membranes and fin rays; two sets of large, dark brown spots on fin rays, first at about their mid-length and second closer to outer membrane; large spots in pairs on 4^th^–8^th^ fin rays; remaining rays with single spots; no large spots centrally on the stout 5^th^ pectoral fin ray; dark brown spots basally on 3^rd^–5^th^ pectoral rays. Tail cream dorsally and laterally with small reddish-brown spots; dark brown blotch centrally on anterior part of tail ring I; clusters of larger spots or blotches forming 4 dark saddles, first on tail rings III–V at about position of dorsal-fin base, second on tail rings VII–VIII, third on tail ring IX–X, and last on tail ring X; some less distinct saddles or groupings of spots present. Dark brown spots present on lateral side of caudoventral plates 1–3 and 6–7. Dorsal and anal fins translucent; each dorsal-fin ray with a small dark brown spot near base and near tip; each anal-fin ray with a small dark spot distally. Caudal fin translucent with dark brown blotch at base and dark brown spots on rays, most evident distally. Pelvic rays cream with 2 faint brown blotches toward tip. Ventral surfaces mostly pale cream and whitish, medium-brown at pectoral-fin base and anterior half of head, including mouth; some dorsal saddles visible ventrally through tail rings, especially posteriorly.

In preservative: Reddish brown and cream fade to shades of greyish brown and yellow, respectively. On carapace, pale dorsal ridges less distinct and blending into background colouration; transverse bar less distinct; dorsal lateral plates finely outlined in black. Dorsal plate margins, ventral plate margins posterior to gular plate, and ventral tail margins outlined in light grey. Pectoral and ventral plates darkened to yellowish brown; ventral base of pectoral fins coloured yellow to yellowish brown.

Paratype (CSIRO H 7491–03, male): Prior to preservation (post-thawing, specimen frozen for 10 years). Similar to holotype, but bands of spots on pectoral fins are usually single spots (not paired), except midway along on 4^th^ and 7^th^ rays which are paired. Each dorsal-fin ray with an additional medial small dark brown spot; each anal-fin ray with two small dark spots distally.

Colour variation amongst measured paratypes and rostrum markings from sexed specimens: Some paratypes without rostral markings, and/or single dark brown bar either absent or replaced by parallel spots, and/or when present, remaining markings appear as 4–8 alternating spots along rostrum edges or 3–4 bars. Large spots on pectoral fins occur either paired or singly. Dark brown to black spots below anterior eye and/or below posterior pupil may be absent. Sometimes with 3 pelvic fin spots.

Freshly caught specimen (paratypes CSIRO H 6543–04 and CSIRO H 7678–01, both males): Carapace including supraorbital tinged olive to dark green; pectoral fins and dorsal tail rings with small orange-brown spots; single orange-brown spot on base of 1^st^ dorsal ray visible in dorsal view; single orange-brown spots anteriorly on tail rings I and II along both dorsal tail ridges.

### Size

Type specimens ranged from 32 to 123 mm PCL (morphometric types ranged from 79 to 98 mm SL, 94 to 116 mm PCL). Two non-type specimens of 11 and 12 mm PCL are probably this species.

### Distribution

*Pegasus tetrabelos* is known from the east coast of Queensland and Torres Strait between latitudes 9°15’ S and 22°01’ S, and in the Northern Territory from the Beagle Gulf to off Darwin (Fig 8). Specimens collected during the Torres Strait and Great Barrier Reef trawl surveys [27,28] formed three distinct clusters (Fig 9). The first cluster was located in the Torres Strait between latitudes 9°15’ S and 10°51’ S; the second cluster in Princess Charlotte Bay (~14°15’ S); and the third from Bowling Green Bay (19°26’ S) south to Broad Sound (22°01’ S). Despite intensive trawling, no specimens of the new species (or *P*. *volitans*) were caught between these clusters in the trawl survey. This likely reflects specific habitat preferences. There are currently no records of this species from the Gulf of Carpentaria or Western Australia.

Specimens of *P*. *tetrabelos* were collected at depths of 8–45 m, mostly in depths less than 30 m. Although the trawling surveys of the Great Barrier Reef and Torres Strait were conducted to depths of 100 m, *P*. *tetrabelos* was not collected in depths greater than 40 m.

### Etymology

The species name *tetrabelos* is a combination of the Greek ‘*tetra’* meaning four and ‘*belos*’ meaning dart or arrow in allusion to the four backward pointing spines on the terminal tail ring (two on each side). The name is treated as a noun in apposition.

### Vernacular names

Short-spined Seamoth.

### Genetic analyses

The genetic identity of the *Pegasus* specimens was based here on their *16S* and *COI* sequence information. Each of the 24 samples sequenced successfully for both mtDNA fragments; average *COI* sequence was 583 base pairs, while the average *16S* sequence was 546 base pairs. The 14 *P*. *volitans* samples were all highly matched (99.7% pairwise identity) to Accession No. AP005984 (*P*. *volitans* mtDNA complete genome, [31]) while the nine *P*. *tetrabelos* samples matched the same Accession (No. AP005984) at a lower level of 97.3% pairwise identity.

Average *16S* nucleotide composition across the *P*. *volitans* specimens consisted of 23.6% Thymine, 23.5% Cytosine, 30.9% Adenine and 22.0% Guanine residues. This was essentially the same across the *P*. *tetrabelos* specimens with the exception of a higher Cytosine content (24.2%). For *COI*, the residue content varied slightly more between the species (*P*. *volitans*: 27.4% T, 27.8% C, 26.1% A, 18.7% G; *P*. *tetrabelos*: 27.6% T, 27.9% C, 25.0% A, 19.5% G).

Evolutionary divergence over pairs of *P*. *volitans* samples (i.e. average genetic distances/diversity from the number of base substitutions per site, ±SE) was 0.001 (±0.001) for *16S* and 0.011 (±0.003) for *COI*. In *P*. *tetrabelos*, the 16S genetic diversity among specimens was also low at 0.001 (±0.000), with a slightly higher divergence of 0.004 (±0.001) for *COI*. The K2 divergence between the two species at the *16S* gene was 0.015 (±0.004), while the *COI* gene reflected a much higher divergence of 0.081 (±0.011). This concordant, yet higher sequence divergence between the two species in the two mtDNA genes was observed in the longer branch lengths of the ML trees for *COI* as compared to *16S* (see Fig 10a and 10b). As Fig 10 shows, there was clear delineation of the species. The *P*. *tetrabelos* sequences clustered together, and specimens of the two *Pegasus* species formed separate groups (with external branch lengths well supported by high bootstrap values). Both species were definitively separated from the outgroup specimen, *E*. *draconis* (Accession No. AP005983). The NJ and Bayesian trees while not shown here, displayed the same topology.

## Discussion

### Comparisons with other species

**Fig 11:**
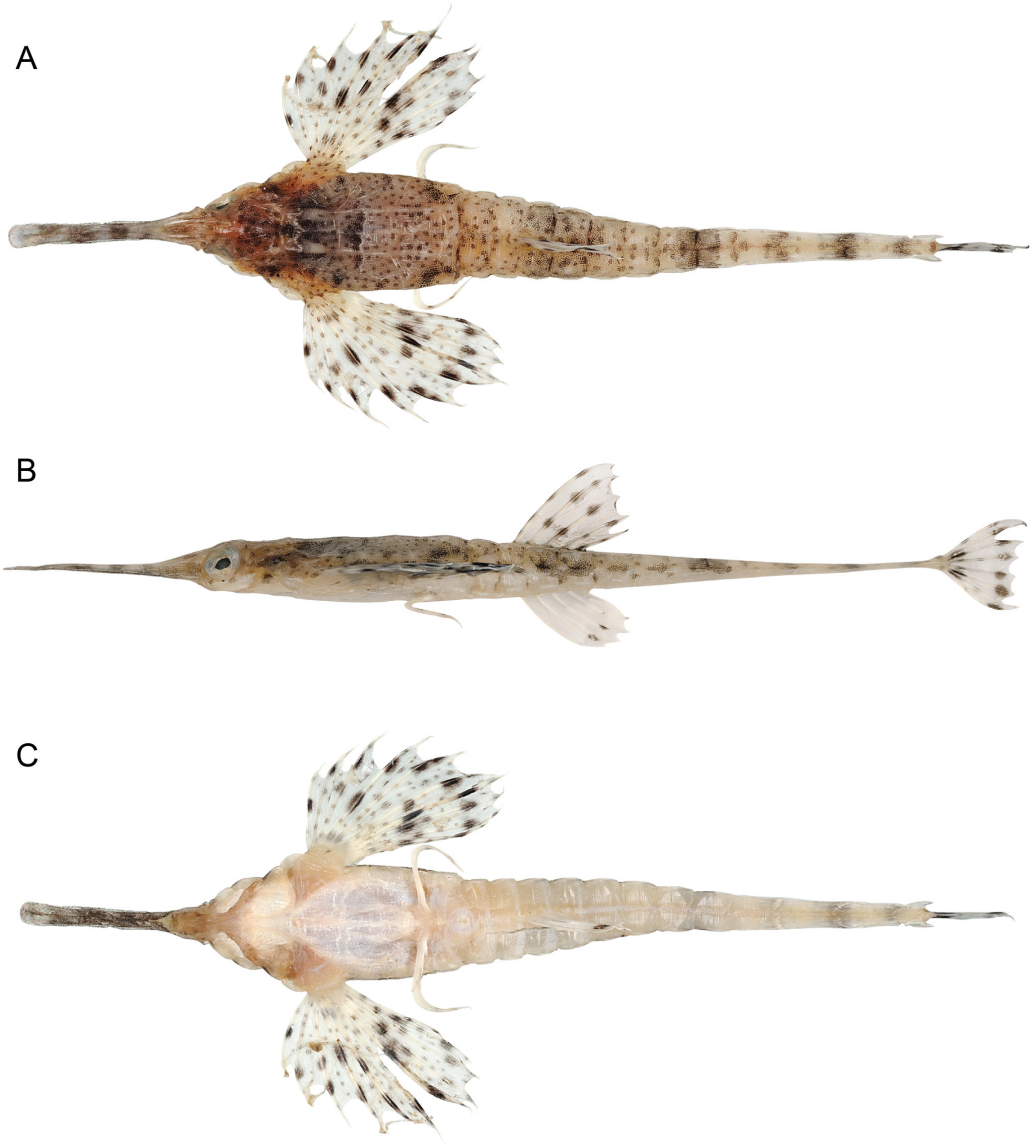
*Pegasus volitans* (CSIRO H 7665–02, 116 mm PCL). (A) dorsal; (B) lateral; and (C) ventral views.

*Pegasus tetrabelos* most closely resembles *P*. *volitans* (Fig 11) and they differ from their congeners *P*. *lancifer* and *P*. *laternarius* in: the number of tail rings (12 vs. 14 and 11, respectively); the number of posteriormost tail rings which are fused together (3 vs. 7 in *P*. *lancifer* and only 9^th^ and 10^th^ tail ring in *P*. *laternarius*); a more slender body (carapace width 13.5–18.1% SL in *P*. *tetrabelos* and 12.8–15.5% SL in *P*. *volitans* vs. 21.3–28.1% SL in *P*. *lancifer* and 25.6–35.8% SL in *P*. *laternarius*; interpectoral width 14.1–17.6 and 12.7–15.5% SL vs. 23.8–28.8 and 33.2–48.7% SL, respectively); and typically with a longer rostrum (rostrum length 22.5–29.5 and 21.3–29.3% SL vs. 6.9–15.1 and 7.8–19.3% SL, respectively). *Pegasus tetrabelos* shares similarities with its congeners *P*. *lancifer* and *P*. *laternarius* in possessing dorsal and ventral pairs of posteriorly-directed spines on the terminal tail ring, and posteriorly-directed tubercles on its dorsal ridge, though less pronounced in *P*. *tetrabelos*. In addition, both *P*. *tetrabelos* and *P*. *laternarius* have a stout 5^th^ pectoral-fin ray, and both *P*. *tetrabelos* and *P*. *lancifer* have a single ventral preopercular notch

**Fig 12:**
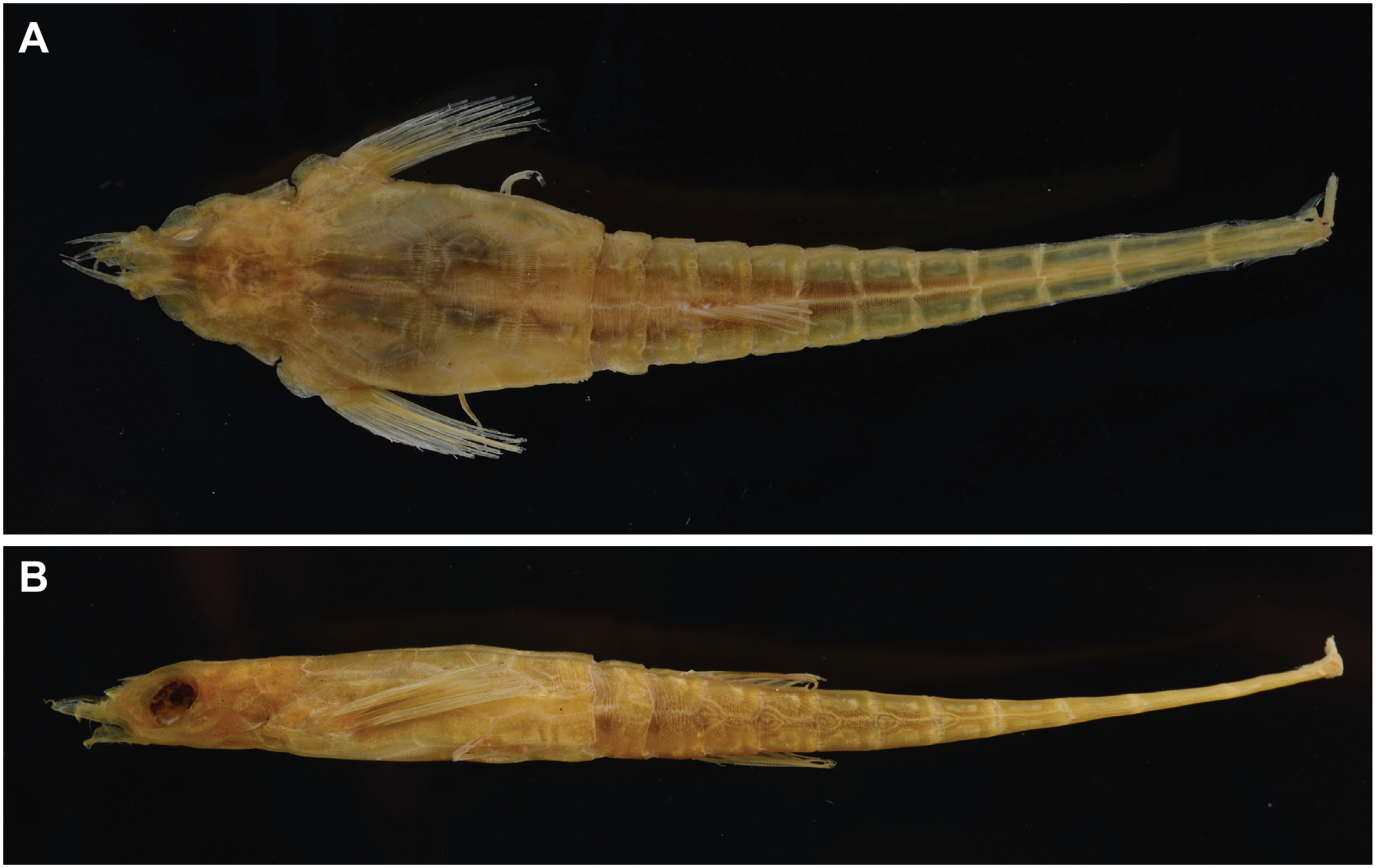
Lectotype of *Pegasus volitans* (NRM LP 30, 108 mm SL). (A) dorsal; and (B) lateral views.

Comparison of the *Pegasus volitans* lectotype (NRM LP 30) with the new species was limited due to its fragile nature and damaged rostrum (Fig 12). It differed from *P*. *tetrabelos* in: length of the terminal tail ring spine when viewed dorsally (c. 4.5 vs. 1.9–3.0% SL); pectoral-fin ray count (11 vs. 10, from radiograph); and presence of a double ventral preopercular notch (vs. single ventral preopercular notch).

*Pegasus tetrabelos* differed from *P*. *volitans* (based on material examined in this study) in the following characters: terminodorsal-lateral and terminoventral-lateral plates with anteriorly and posteriorly directed spines present (vs. absent in *P*. *volitans*), terminal-lateral plates absent (vs. present, see Fig 7); shorter tail ring XII spine (1.9–3.0 vs. 4.1–5.5% SL), spines on lateral tail ridges demarcate earlier (posterior tail ring IX vs. posterior tail ring X–XI or not demarcating, see Fig 6); wider tail ring X (width 4.1–5.0 vs. 3.4–4.0% SL), 4 dark caudal saddles, no prominent saddle on tail ring XI (vs. 5 dark caudal saddles including prominent saddle on tail ring XI, see Figs 2 vs. 11); pectoral fin rays usually 10 (vs. usually 11); 5^th^ pectoral-fin ray stout (vs. not stouter than adjacent rays); single ventral preopercular notch (vs. double ventral preopercular notch, see Fig 4); carapace with three small posteriorly directed tubercles along each dorsal ridge, one at the centre of each dorsal plate (vs. no posteriorly directed tubercles, see Fig 3). Lateral keels are often comparatively more defined and posteriorly-directed in *P*. *tetrabelos* (see Fig 5).

*Pegasus tetrabelos* and *P*. *volitans* were frequently caught together in trawls at the same depths on sandy or muddy substrata in the Great Barrier Reef and Torres Strait regions. Further research is required to determine whether these two species share the same habitats or whether there are fine-scale habitat preferences between them. *Pegasus tetrabelos* appears to have a much more restricted distribution than *P*. *volitans*. However, closer examination of *P*. *volitans* specimens from various locations throughout its wide Indo-West Pacific range is required to determine whether only a single species is involved or whether there are further cryptic speciation issues which need to be resolved.

Although the whereabouts of the types of *Pegasus pristis* [24] are unknown, it can be ruled out as an available name for *P*. *tetrabelos* based on the number of pectoral-fin rays recorded in the description, i.e. 11 (9 or 10 in *P*. *tetrabelos*). Furthermore, Bleeker’s description mentions that both sides of the last caudal ring with a very conspicuous sharp thorn which aligns with the *P*. *volitans* rather than *P*. *tetrabelos* which possesses two sharp thorns on each side which are less conspicuous. As outlined previously, *Cataphractus anceps* of [26] was based on the same source, i.e. [21]), as used by [23] for *P*. *natans*, with no types known. The illustrations of this species in [21] clearly show a large and very distinct spine on the terminal tail ring (Fig 1), far larger than present in *P*. *tetrabelos*. Since *P*. *volans* is based on NRM LP 30 which is the lectotype of *P*. *volitans*, the above available names (*P*. *anceps*, *P*. *natans*, *P*. *pristis*, and *P*. *volans*) remain in the synonymy of *P*. *volitans*.

### DNA barcoding comparisons

The aim of the DNA barcoding investigation here was not to undertake a comprehensive phylogenetic study of the Pegasidae family, but rather focus on whether a species complex may exist within *P*. *volitans* in Australian waters. As shown in this study, samples nominally identified as *P*. *tetrabelos* were clearly separated from *P*. *volitans*. Supported by the integrated taxonomic approach, the DNA barcoding of two mtDNA genes strongly supports the presence of both *P*. *volitans* and *P*. *tetrabelos* specimens. Additionally, while both genes detected species level sequence differences, the genetic divergence in the *COI* gene was higher than the *16S* gene. Both genes identified a higher level of within species variation present in the *P*. *volitans* samples. This was evident by two sub-groups (with highest bootstrap support in the COI gene) observed in the *P*. *volitans*.

### Intraspecific variation

Intraspecific variations in *Pegasus tetrabelos*, which are largely provided in the description section, include morphological characters and aspects of the coloration. Variations in rostral and pectoral fin markings, and variations in rostral shape (e.g. tip club shaped vs. not club shaped) could not be consistently attributed to differences in sex, size (as a proxy for age), or region. Genetic analyses of the *16S* and *COI* mtDNA genes also detected low levels of genetic variation present in the nine *P*. *tetrabelos* specimens that were barcoded.

Examination of specimens sexed via dissection revealed morphological characters which differed between female and male *Pegasus tetrabelos*. Consistently differing characters were trunk width and maximum rostrum width. Females had wider trunk widths (16.2–19.6 vs. 13.1–15.7% SL), and narrower maximum rostrum widths (4.8–5.4 vs. 6.2–6.7% SL). These sexually dimorphic characters were only apparent in specimens greater than approximately 74 mm SL, with females and males less than 63.5 mm SL not differing in these characters. Thus, these characters are possibly a proxy for an approximate size at maturity in this species.

### Conservation and management implications

This study has delineated and described a new cryptic species, *Pegasus tetrabelos*, from what was previously a commonly-recognised, wide-ranging species. This has been strongly supported by multiple taxonomic tools through an integrated taxonomy approach, and will provide a basis for scientific studies, and informed management and conservation efforts into the future.

A consequence of having a single wide-ranging species split into two, with one species having a much more restricted range, is that there is a need to reassess management or impacts relevant to each species. *Pegasus* species are caught as bycatch in northern Australian commercial prawn trawl fisheries, where they are discarded [32]. The abundance of ‘*P*. *volitans*’ was reported to have declined in the southeast Gulf of Carpentaria after 20 years of prawn trawl fishing, with demersal fish surveys recording a reduction in catches of 32% [33]. In the East Coast Trawl Fishery and Torres Strait Prawn Fishery, ‘*P*. *volitans*’ were found to be caught in ~24 and 41% of prawn trawls, respectively [34,35]. Although it is not possible to determine the relative contribution of *P*. *volitans* and *P*. *tetrabelos* from this data, it is likely both species are encountered in these two fisheries.

Although pegasids are not utilised in large quantities in Australian waters, there is evidence of far greater exploitation of pegasids in some Asian countries. In a single Philippine province, it is estimated 43,000–62,000 seamoths/year (predominantly *P*. *volitans*) are caught live for the aquarium trade, with an additional 130,000–620,000 *P*. *volitans*/year caught incidentally by fishers and sold to traditional Chinese medicine markets [36]. Sales of *P*. *volitans* for traditional medicine in China are estimated to be in the millions each year, provided by suppliers throughout Southeast Asia [12]. This highlights the need for further taxonomic work on this group outside of Australia. If the wide-ranging *P*. *volitans* is found to be a complex of species with more restricted ranges, such localised heavy exploitation could be a more significant threat than currently recognised.

## Supporting Information

S1 Text Comparative material. Collection data for the comparative material examined in this study.
(DOCX)

S2 Text DNA barcoding. DNA extractions and sequencing analyses
(DOCX)

S1 Fig Morphological measurements taken from *Pegasus* specimens. Dorsal, lateral and ventral illustrations of a stylised *Pegasus volitans* illustrating the measurements used in this study.
(TIF)

S2 Fig Configuration of external plates in *Pegasus volitans*. (A) dorsal and (B) ventral views taken from [37]. Abbreviations for head, body and tail plates (all plates are paired except where noted) taken from [14]: a = anal plate; ca.d. = dorsal ridge; ca.dl. = dorsolateral ridge; ca.vl. = ventrolateral ridge; cd_1–11_ = caudodorsal plates; cl_l–6_ = caudolateral plates; ct = cleithrum; cv_1–11_ = caudoventral plates; d_1–3_ = dorsal plates; dl_l–4_ = dorsolateral plates; ect = ectethmoid; f.p.v. = ventral-fin foramen; fr = frontal; g = gular plate; ip = interpectoral plate (unpaired); iv = interventral plate (unpaired); met = mesethmoid (unpaired); na = nasal (paired elements fused on midline); p = pectoral plate; pa = preanal plate (unpaired); pp.s. = superior pectoral-fin plate; pp.i. = inferior pectoral-fin plate; td = terminal-dorsal plate (unpaired); tl = terminal-lateral plate; tv = terminal-ventral plate (unpaired); v = ventral plate; vl_l–5_ = ventrolateral plates.
(TIF)

S1 Table Morphological methodology. Description of the morphological characters used in this study (see also S1 Fig).
(XLSX)

S2 Table Genetic sample information. (XLSX)
