# Correction: Integrated Taxonomy Reveals Hidden Diversity in Northern Australian Fishes: A New Species of Seamoth (Genus *Pegasus*)

**DOI:** 10.1371/journal.pone.0251680

**Published:** 2021-05-07

**Authors:** Deborah Osterhage, John J. Pogonoski, Sharon A. Appleyard, William T. White

In Fig 10, the COI GenBank Accession Numbers for the *Pegasus* species individuals in Plate B are incorrect. Please see the correct Fig 10 here.

**Fig 10:**
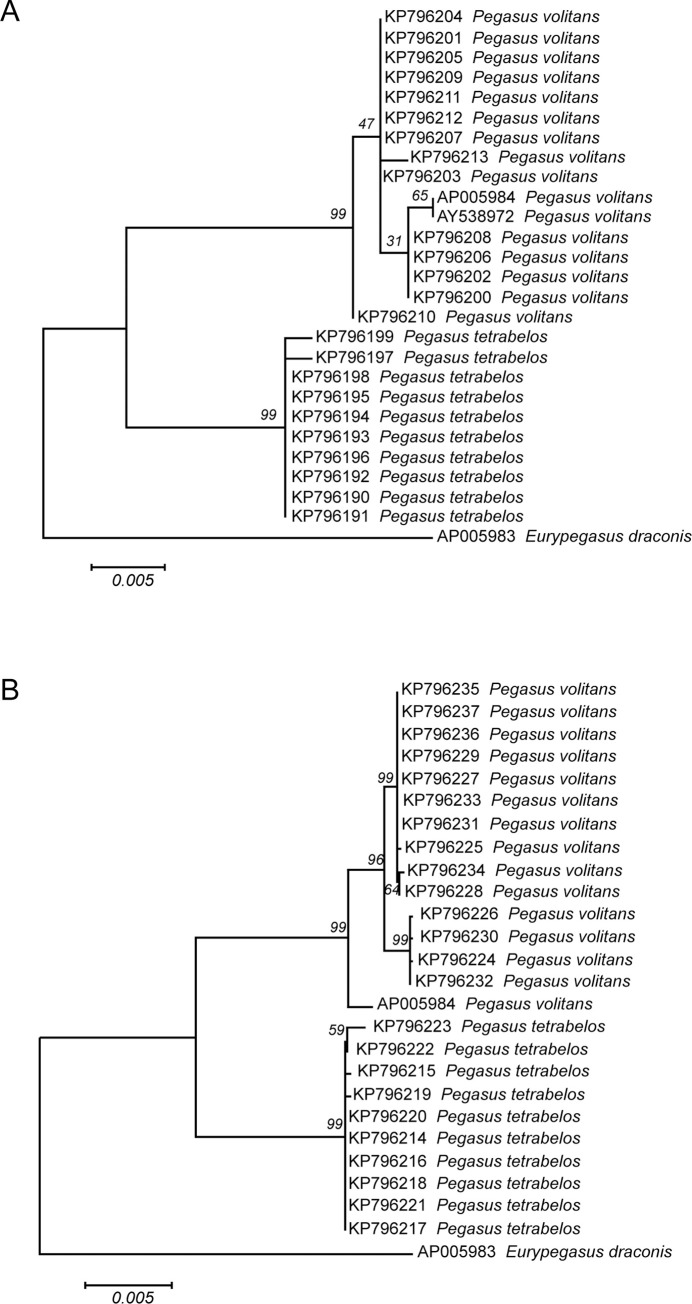
Molecular species identification of *Pegasus* species using Genetic treeML trees. (A) sequences from the *16S* gene; (B) sequences from the *COI* gene. Trees are based on the K2 evolutionary distance model and are shown here with mined *Pegasus* and *Eurypegasus* sequences from GenBank. The trees are shown here with an *E*. *draconis* outgroup. Bootstrap support values (following 1000 replicates) are shown above the nodes.

The COI GenBank Accession Numbers for the *Pegasus* species individuals in S2 Table are incorrect. Please view the correct S2 Table below.

## Supporting information

S2 Table Genetic sample information. (XLSX)
