# Supplementary material for: Integrated Taxonomy Reveals Hidden Diversity in Northern Australian Fishes: A New Species of Seamoth (Genus Pegasus)
Source: PLoS One. 2016 Mar 2;11(3):e0149415. doi: 10.1371/journal.pone.0149415 (PMC4774964; doi:10.1371/journal.pone.0149415)
Supplement: S1 Text — Collection data for the comparative material examined in this study. (DOCX) [file pone.0149415.s001.docx]

**S1 Text. Comparative material.** List of *Pegasus* specimens examined during this study.

*Pegasus lancifer*

Tasmania, Australia: CSIRO A 4325, 60 mm PCL, Babel Island, 39°57’ S, 148°20’ E, 6 Oct 1977; CSIRO H 677-1, 76 mm PCL, north of Stony Head, 40°56.8’ S, 147°01.3’ E, 24 m depth, 17 Aug 1986; CSIRO H 4724-01, 66 mm PCL, Nutgrove Beach, Derwent River, ~42°55’ S, 147°21’ E, 29 Mar 1988; CSIRO T 1074, 71 mm PCL, Green Island, 43°12’ S, 147°17’ E, 1 Jun 1973; CSIRO T 1149 (3 specimens), 57–70 mm PCL, Bass Strait; CSIRO T 1172, 63 mm PCL, Coles Bay, ~42°08’ S, 148°17’ E, 4 Apr 1980; CSIRO T 1653, 31 mm PCL, Nutgrove Beach, Derwent River, ~42°55’ S, 147°21’ E, 28 Feb 1977.

Victoria, Australia: CSIRO A 1662, 69 mm PCL, Lakes Entrance, ~37° 53’ S, 148° E, 54 m depth, 27 Jan 1953.

No location: CSIRO A 1055, 64 mm PCL; CSIRO T 1142 (2 specimens), 84–85 mm PCL.

*Pegasus volitans*

**Lectotype**. NRM LP 30 (also holotype of *Pegasus volans*), 108 mm SL, no collection location or date.

**Other material**. New South Wales, Australia: AMS IA. 30, 57 mm PCL, Tuggerah Lakes, 33°18’ S, 151°30’ E, 1920; AMS IA. 1849, 49 mm PCL, Shell Harbour, 34°35’ S, 150°52’ E, 1924; AMS IA. 5100, 98 mm PCL, Lake Macquarie, 33°02’ S, 151°36’ E, 13 Apr 1912; AMS IA. 5483, 98 mm PCL, Narrabeen, Sydney, 33°43’ S, 151°18’ E, 1932; AMS IA. 6360, 67 mm PCL, Northern Rivers, 1935; AMS IB. 1471, 98 mm PCL, Newcastle, 32°57’ S, 151°45’ E, 1946; AMS IB. 2065, Lake Macquarie, 33°28’ S, 151°26’ E, 10 Apr 1948; AMS IB. 2209, 71 mm PCL, Nambucca Heads, 30°39’ S, 153°01’ E, 1949; AMS IB. 3482, Wommin Lagoon, Tweed River, 28° S, 153° E, 1956; AMS IB. 3570, Narrabeen, Sydney, 33°43’ S, 151°18’ E, Jun 1956; AMS IB. 3654, 74 mm PCL, off Port Stephens, 32° S, 152° E, 73 m depth, 29 Oct 1956; AMS IB. 4214, Mackerel Beach, Bondi, Sydney, 33°53’ S, 151°17’ E, 1959; AMS IB. 5077, Laurieton, 31°39’ S, 152°48’ E, 1961; AMS IB. 5987, The Entrance, Tuggerah, 33°21’ S, 151°30’ E, Mar 1960; AMS IB. 7084, Long Bay, Rock Baths, 33°58’ S, 151°15’ E, Mar 1964; AMS IB. 7266, Broken Bay, 33°34’ S, 151°20’ E, 9 m depth, 1965; AMS IB. 7297, 70 mm PCL, Port Jackson, 33°51’ S, 151°16’ E, 3 Feb 1965; AMS I. 16805-001, 70 mm PCL, Minnamurra Estuary, 34°35’ S, 150°55’ E, Feb 1967; AMS I. 17747-001, 36 mm PCL, Bottle and Glass Rocks, Sydney, 33°51’ S, 151°16’ E, 15 Mar 1974; AMS I. 20017-001, 36 mm PCL, East Gladesville Bridge, Parramatta River, 33°51’ S, 151°08’ E, 9 Dec 1977; AMS I. 38608-001, 100 mm PCL, south east of Yamba, 29°38’ S, 153°20’ E, 18 m depth, 10 Apr 1996; AMS I. 41290-001, 20 mm PCL, Harbour Beach, Iluka, 29°24’28” S, 153°20’55” E, 1.5 m depth, 26 Mar 2002.

Northern Territory, Australia: AMS I. 24687-010, 36 mm PCL, East Arm, Darwin, 12°29’ S, 130°54’ E, 1 m depth, 11 Sep 1984; CSIRO A 2632, 108 mm PCL, CSIRO A 2633, 107 mm PCL, Arnhem Bay, 12°20’ S, 136°10’ E, 11–27 m depth, 1961; CSIRO A 2633, 103 mm PCL, Melville Bay, 12°15’ S, 136°43’ E, 3 m depth, 2 Jul 1960; CSIRO C 4242, 131 mm PCL, 17 miles northeast of Robinson River, Gulf of Carpentaria, 15°55’ S, 137°30’ E, 3 m depth, 30 Oct 1971; CSIRO CA 2671, 106 mm PCL, north of Groote Eylandt, Gulf of Carpentaria, 13° S, 136° E, 3 m depth, 9 Jul 1981; NTM S.11722-010, 53 mm PCL, Woods Inlet, Darwin Harbour, 12°31’01” S, 130°44’10” E, 30 Aug 1985; NTM S.12769-001, 47 mm PCL, west of Nightcliff boat ramp, Darwin Harbour, 12°22’01” S, 130°49’48” E, 9 m depth, 3 Mar 1989; NTM S.13723-005, 22 mm PCL, mouth of Hudson Creek, Darwin Harbour, 12°29’16” S, 130°55’22” E, 2 m depth, 16 Jul 1993; NTM S.00499-001, 97 mm PCL, NTM S.00500-001, 96 mm PCL, off Christmas Creek, Gulf of Carpentaria, 14°30’00” S, 141°31’01” E, 5 Mar 1976; NTM S.10153-001 (2 specimens), 34–62 mm PCL, Shoal Bay, Darwin Harbour, 12°20’24” S, 130°58’58” E, 9 Oct 1973; NTM S.10156-001, 105 mm PCL, east of Micket Creek mouth, Shoal Bay, Darwin Harbour, 12°20’41” S, 130°58’22” E, 25 Jul 1973; NTM S.10283-001 (3 specimens), 52–68 mm PCL, reefs off Buffalo Creek, Shoal Bay, Darwin Harbour, 12°20’ S, 130°55’01” E, 8 Sep 1972; NTM S.10429-022, 5 mm PCL, Channel Island, north side, Darwin Harbour, 12°33’02” S, 130°52’01” E, 24 May 1982; NTM S.11949-002, 27 mm PCL, off Caiman Creek, Port Essington, 11°13’58” S, 132°10’58” E, 2 m depth, 12 Sep 1985; NTM S.13724-008, 15 mm PCL, off Pearl Raft Creek, Darwin Harbour, 12°31’01” S, 130°54” E, 3 m depth, 16 Jul 1993; NTM S.13779-001, 115 mm PCL, northwest of Roche Reef, Beagle Gulf, 12°31’01” S, 130°13’58” E, 19 m depth, 5 Oct 1993; NTM S.13791-001, 112 mm PCL, northwest of Charles Point, Beagle Gulf, 12°19’01” S, 130°34’01” E, 20 m depth, 7 Oct 1993; NTM S.15054-001, 54 mm PCL, Ludmilla Creek, Darwin Harbour, 12°24’46” S, 130°50’13” E, 16 Jul 1999; NTM S.15967-002, 23 mm PCL, northwest side of Field Island, Kakadu National Park, 12°05’38” S, 132°19’19” E, 3 m depth, 24 Nov 2004; NTM S.15998-004, 56 mm PCL, southeast of Milingimbi Island, Arnhem Land, 12°08’27” S, 134°56’31” E, 5 m depth, 5 Dec 2004; NTM S.16715-008, 119 mm PCL, off Numbulwar, Groote Eylandt, 14°15’ S, 136°04’58” E, 19 m depth, 24 Sep 1971.

Queensland, Australia: AMS E. 2950, 114 mm PCL, 21 km southeast of Cape Capricorn, 23°35’ S, 151°27’ E, 26 m depth, 29 Jul 1910; AMS I. 501, 105 mm PCL, Moreton Bay, 27°25’ S, 153°20’ E, 1886; AMS I. 504 (2 specimens), 98–104 mm PCL, Moreton Bay, 27°25’ S, 153°20’ E, 1886; AMS IA. 33, Mornington Island, Gulf of Carpentaria, 16°36’ S, 139°21’ E, 1920; AMS IA. 175, 61 mm PCL, Bowen, 20°01’ S, 148°15’ E, 1921; AMS IA. 1696, 93 mm PCL, Young Island, Cumberland Group, 20°45’ S, 149°20’ E, 1923; AMS IA. 2335, Maori Reef, Great Barrier Reef, 17°07’ S, 146°21’ E, 1924; AMS IA. 2336, 85 mm PCL, McCulloch Reef, Great Barrier Reef, 17°18’ S, 146°28’ E, 1924; AMS IA. 7348, 52 mm PCL, Southport, 27°58’ S, 153°25’ E, Oct 1937; AMS IB. 2788, 55 mm PCL, Port Denison, Gregory River, 20°03’ S, 148°15’ E, 1885; AMS IB. 6898, AMS IB. 6899, AMS IB. 6900, AMS IB. 6901, AMS IB. 6902, 125 mm PCL, off Sweers Island, Gulf of Carpentaria, 17°06’ S, 139°37’ E, 9 Dec 1963; AMS IB. 7074, Karumba District, Gulf of Carpentaria, 17°29’ S, 140°50’ E, 1934; AMS I. 15557-084 (5 specimens), 95–102 mm PCL, Gulf of Carpentaria, 17°25’ S, 140°10’ E, 10 m depth, 27 Nov 1963; AMS I. 18657-002, 109 mm PCL, Amity, Moreton Island, 27°24’ S, 153°26’ E, Mar 1975; AMS I. 20771-013 (6 specimens), 122–132 mm PCL, 1–9 mile east of Captain Billy Creek, Cape York, 11°37’ S, 142°56’ E, 16–18 m depth, 18 Feb 1979; AMS I. 22083-006, 38 mm PCL, Norman River entrance, Karumba Point Beach, 17°28’ S, 140°50’ E, 1 m depth, 30 Sep 1980; AMS I. 24200-011, off Townsville, 19°08’ S, 147°03’ E, 1978; AMS I. 34362-003 (2 specimens), 52–61 mm PCL, 1 km south of Entrance Island at entrance to Port Clinton, 22°29’59” S, 150°46’17” E, 9 m depth, 12 Oct 1993; CSIRO A 813, 85 mm PCL, CSIRO A 814, 83 mm PCL, CSIRO A 815, 79 mm PCL, Cowan Cowan, Moreton Island, 27°08’ S, 153°21’ E, 15 Jun 1941; CSIRO A 1031, 101 mm PCL, CSIRO A 1032, 101+ mm PCL (rostrum tip damaged), CSIRO A 1033, 90 mm PCL, CSIRO A 1034, 103 mm PCL, Tangalooma Point, Moreton Island, 27°11’ S, 153°22’ E, 11 m depth, 12 Jun 1950; CSIRO A 1045, 112 mm PCL, CSIRO A 1046, 113 mm PCL, CSIRO A 1047, 110 mm PCL, CSIRO A 1048, 100 mm PCL, CSIRO A 1049, 98 mm PCL, CSIRO A 1050, 97 mm PCL, off Point lookout, Stradbroke Island, 27°26’ S, 153°33’ E, 11 m depth, 6 Jun 1950; CSIRO A 1736, 42 mm PCL, Dunwich Bight, Stradbroke Island, 27°30’ S, 153°24’ E, off beach, 17 Aug 1950; CSIRO A 2246, 107 mm PCL, Gulf of Carpentaria, 17°35’47” S, 140°15’48” E, 4 m depth, 31 Jul 1963; CSIRO A 2369, 106 mm PCL, northwest of Karumba, Gulf of Carpentaria, 17°13’24” S, 140°35’06” E, 11 m depth, 30 Jul 1963; CSIRO A 3183, 101 mm PCL, Gulf of Carpentaria, 16°52’30” S, 140°37’30” E, 16.5 m depth, 24 May 1965; CSIRO A 4100, 104 mm PCL, 30 miles northwest of Fairway Buoy, Gulf of Carpentaria, 16 m depth, 30 Oct 1972; CSIRO CA 2139, 106 mm PCL, east of Bentinck Island, Gulf of Carpentaria, 16°57’ S, 140°11’ E, 16 m depth, 7 Dec 1980; CSIRO H 3607-01, 105 mm PCL, west northwest of Weipa, Gulf of Carpentaria, 12°28’30” S, 141°31’36” E, 18 m depth, 14 Nov 1993; CSIRO H 6146-07, 91 mm PCL, south of Saibai Island, Torres Strait, 09°36’37” S, 142°34’58” E, 11–17 m depth, 23 Jan 2004; CSIRO H 6507-04, female 120 mm PCL, northeast of Bundaberg, 24°17’25” S, 152°51’32” E, 42 m depth, 15 Apr 2004; CSIRO H 6510-03, 127 mm PCL, east of Newcastle Bay, 10°58’21” S, 143°09’37” E, 26 m depth, 26 Sep 2004; CSIRO H 6513-03, 121 mm PCL, northeast of Bundaberg, 24°20’07” S, 153°01’40” E, 48 m depth, 14 Apr 2004; CSIRO H 6548-06, 128 mm PCL, north of Bundaberg, 24°25’45” S, 152°04’22” E, 14 m depth, 13 Apr 2004; CSIRO H 6553-04, 109 mm PCL, northeast of Dungeness Island, Torres Strait, 9°46’38” S, 143°09’33” E, 19 m depth, 24 Jan 2004; CSIRO H 6649-02, 121 mm PCL, east of Bowling Green Bay, 19°23’31” S, 147°29’24” E, 12 m depth, 2 Dec 2003; CSIRO H 6692-02 (3 specimens), 99–117 mm PCL, east of Dungeness Island, Torres Strait, 9°53’37” S, 143°08’03” E, 24 m depth, 29 Jan 2004; CSIRO H 6710-03, 100 mm PCL, north of Townsville, 18°56’55” S, 146°35’05” E, 14 m depth, 11 Dec 2005; CSIRO H 6738-04 (2 specimens), 128–134 mm PCL, CSIRO H 6738-05, 125 mm PCL, southeast of Mackay, 21°17’58” S, 149°34’11” E, 21 m depth, 29 Apr 2004; CSIRO H 6901-05, 117 mm PCL, CSIRO H 6901-06, 103 mm PCL, west of Dungeness Island, Torres Strait, 9°56’45” S, 142°53’17” E, 10 m depth, 20–21 Jan 2004; CSIRO H 6903-03, 108 mm PCL, CSIRO H 6903-04, female 111 mm PCL, west of Dungeness Island, Torres Strait, 9°51’08” S, 142°45’05” E, 15 m depth, 21 Jan 2004; CSIRO H 6914-03, 115 mm PCL, west of Mulgrave Island, Torres Strait, 10°02’43” S, 141°36’32” E, 16 m depth, 16 Jan 2004; CSIRO H 7665-02, 116 mm PCL, north of Dalrymple Island, Torres Strait, 9°21’55” S, 143°24’18” E, 20 m depth, 25 Jan 2004; CSIRO H 7669-01 (2 specimens), 124–125 mm PCL, south of Dalrymple Island, Torres Strait, 9°43’32” S, 143°15’30” E, 22 m depth, 24 Jan 2004; CSIRO H 7671-01 (6 specimens), 114–126 mm PCL, northeast of Newcastle Bay, Torres Strait, 10°24’01” S, 143°04’51” E, 23 m depth, 11 Jan 2004; CSIRO H 7672-01 (4 specimens), 81–104 mm PCL, south of Saibai Island, Torres Strait, 9°35’54” S, 142°50’55” E, 9 m depth, 23 Jan 2004; CSIRO H 7673-01, 116 mm PCL, east of Banks Island, Torres Strait, 10°06’44” S, 142°39’38” E, 17 m depth, 12 Jan 2004; CSIRO H 7674-02, male 122 mm PCL, east of Flinders Group, 14°10’48” S, 144°11’38” E, 15 m depth, 21 Sep 2004; CSIRO H 7692-01 (1 male, 1 female), 124–128 mm PCL, north of Cape Hillsborough, 20°49’46” S, 149°06’16” E, 16 m depth, 10 Dec 2003; CSIRO H 7693-01, male 125 mm PCL, south of Dalrymple Island, Torres Strait, 9°41’05” S, 143°21’41” E, 25 m depth, 24 Jan 2004; NRM 66670, 120 mm PCL, northeast of Cocoanut Island, Torres Strait, 10°01’23” S, 143°13’34” E, 26 m depth, 29 Jan 2004; QM I.8503 (1 specimen), 126 mm PCL, off Weipa, Gulf of Carpentaria, ~12°37’ S 141°52’ E, 7.3–9.1 m depth, 2 Oct 1961; QM I.20900 (2 specimens), 115-122 mm PCL, Gulf of Carpentaria, 16°39’ S 139°40’ E, 18 m depth, 18 Jan 1983; QM I. 36490 (1 specimen), 99 mm PCL, NW of Yellow Patch, Moreton Island, 26°58’ S, 153°25’ E, 18 m depth, 16 Feb 2005; QM I.37907 (4 specimens), 112–129 mm PCL, 24°20’06” S, 152°32’ E, 33m depth, 6 Nov 2005; USNM 434843, 119 mm PCL, north of Cape Hillsborough, 20°49’25” S, 149°05’48” E, 16 m depth, 10 Dec 2003; WAM P. 34326-001, 115 mm PCL, Princess Charlotte Bay, 14°19’46” S, 143°55’04” E, 8 m depth, 3 Mar 2006; WAM P. 281-001, 92 mm PCL, 11 km south of Double Island Point, 25°56’ S, 153°11’ E, 58.5 m depth; WAM P. 5216-001, 97 mm PCL, Albatross Bay, Gulf of Carpentaria, 12°40’ S, 141°42’ E, 6.4 m depth, 20 Jan 1962; WAM P. 5627-001 (2 specimens), 98–125 mm PCL, lighthouse at Cape Bowling Green, 19°20’ S, 147°26’ E, 23 Nov 1962; WAM P. 5628-001, 93 mm PCL, 0.8 km offshore of Weipa, Gulf of Carpentaria, 12°39’ S, 141°53’ E, 6 m depth, 10 Mar 1962; WAM P. 6259-001, 65 mm PCL, Weipa, Gulf of Carpentaria, 12°39’ S, 141°53’ E, Nov 1961; WAM P. 10822-001, Gulf of Carpentaria, 14° S, 140° E, 1964; WAM P. 11847-001, 111 mm PCL, Gulf of Carpentaria, 14° S, 140° E, 1964; WAM P. 12786-001, 117 mm PCL, Gulf of Carpentaria, 15° S, 140° E, Nov 1964; WAM P. 28351-003, 110 mm PCL, Abbott Point, Bowen, 19°53’ S, 148°05’ E; WAM P. 2877-026, 115 mm PCL, Moreton Bay, 27°15’ S, 153°15’ E, 1972; WAM P. 28823-001, 38 mm PCL, Tagalooma, Moreton Bay, 27°12’ S, 153°22’ E, 0.1–6 m depth, 5 Mar 1973; WAM P. 28828-008, 110 mm PCL, Welsby, Moreton Bay, 27°06’ S, 153°20’ E, 1.5–3 m depth, 1972.

Western Australia, Australia: AMS IA. 4134, 114 mm PCL, AMS IA. 4135, southwest of Cape Jaubert, 19° S, 121° E, 9 m depth, Sep 1929; AMS IA. 4232, between Broome and Cape Bossutt, 18° S, 121° E, 9 m depth, Sep 1929; AMS IA. 5114, Broome, 17°58’ S, 122°14’ E, 1931; AMS IB. 8244, Exmouth Gulf, 21°53’ S, 114°15’ E, 13–18 m depth, 9 Aug 1967; AMS I. 13260 (2 specimens), 85–95 mm PCL, Swan River, 32°01’ S, 115°48’ E, 1914; AMS I. 20788-012, 110 mm PCL, off Carnarvon, 24°53’ S, 113°40’ E, May 1972; AMS I. 33311-015 (2 specimens), 91–100 mm PCL, Exmouth Gulf, 22°05’ S, 114°15’ E, 21 m depth, 12 Sep 1972; CSIRO A 1323, 91 mm PCL, CSIRO A 1324, 104 mm PCL, Cockburn Sound, 12 Mar 1954; CSIRO A 1416, 99 mm PCL, Dampier Archipelago, 20°32’ S, 116°35’ E, 1954; CSIRO A 3368, 96 mm PCL, Tent Point, Exmouth Gulf, 21°59’39” S, 114°30’37” E, 17 Sep 1966; CSIRO B 1983 (5 specimens), 112–118 mm PCL, east of Stewart Island, 20°53’ S, 115°58’ E, 7 Dec 1979; QM I. 14267 (2 specimens), 103–118 mm PCL, Dampier, 1977; WAM P. 50-001, 70 mm SL, Fremantle, 32°03’ S, 115°44’ E; WAM P. 607-001, 67 mm PCL, Swan River, 32°01’ S, 115°48’ E, 1918; WAM P. 633-001, 78 mm PCL, Swan River, Attadale, 32°01’ S, 115°48’ E, 1918; WAM P. 692-001, 43 mm PCL, Garden Island, 32°12’ S, 115°40’ E, 1920; WAM P. 1110-001, 68 mm PCL, Fremantle, 32°03’ S, 115°44’ E, 1930; WAM P. 1111-001, 82 mm PCL, Swan River, 32°01’ S, 115°48’ E, 1930; WAM P. 1168-001, 73 mm PCL, South Beach, Fremantle, 32°03’ S, 115°44’ E, 1931; WAM P. 1414-001, 47 mm PCL, Shark Bay, 25°21’ S, 113°44’ E, 27 Feb 1935; WAM P. 1435-001, 118 mm PCL, Freshwater Bay, 32°01’ S, 115°48’ E, 1935; WAM P. 2206-001 (dried), 68 mm PCL, 32°01’ S, 115°48’ E, 1940; WAM P. 2259-001 (dried), 87 mm PCL, 32°01’ S, 115°48’ E, 1940; WAM P. 2296-001 (dried), 92 mm PCL, 32°01’ S, 115°48’ E, 1941; WAM P. 3292-001 (dried), 81 mm PCL, 32°01’ S, 115°48’ E, 1950; WAM P. 3454-001 (dried, 2 specimens), 93–99 mm PCL, Geraldton, 28°46’ S, 114°37’ E, 11 Sep 1951; WAM P. 3963-001 (dried), 110 mm PCL, Fremantle Harbour, 32°03’ S, 115°44’ E, May 1956; WAM P. 4271-001, 127+ mm PCL (rostrum tip damaged), between Koks Island and Point Quobba, 24°37’ S, 113°17’ E, 23 Jul 1958; WAM P. 5255-001, 119+ mm PCL (rostrum tip damaged), Shark Bay, 25°21’ S, 113°44’ E, Jun 1962; WAM P. 5263-001, 117+ mm PCL (rostrum tip damaged), north of Peron Flats, Shark Bay, 25°29’ S, 113°31’ E, Jul 1962; WAM P. 5477-001 (2 specimens), 119–124 mm PCL, 64 km south of Carnarvon, 25°30’ S, 113°40’ E, Jun 1960; WAM P. 5843-001, 115+ mm PCL (rostrum tip damaged), Shark Bay, 25°21’ S, 113°44’ E, Jul 1963; WAM P. 5908-001, 50 mm PCL, Penguin Point jetty, Safety Bay, 32°18’ S, 115°43’ E, 26 Jan 1964; WAM P. 5976-001 (dried), 78 mm PCL, Nedlands, 32°00’ S, 115°44’ E, 8 Apr 1964; WAM P. 5977-001 (dried), 66 mm PCL, North Mole, Fremantle, 32°03’ S, 115°44’ E, 16 Mar 1964; WAM P. 6222-001, 37 mm PCL, Cockburn Sound, 32°11’ S, 115°43’ E, 10 Jan 1959; WAM P. 6277-001, 121 mm PCL, Shark Bay, 25°21’ S, 113°44’ E, 25 Feb 1962; WAM P. 8919-001, 118 mm PCL, Shark Bay, 25°21’ S, 113°44’ E, 14.6 m depth, 10 Oct 1964; WAM P. 9130-001, 105 mm PCL, Shark Bay, 25°21’ S, 113°44’ E, 14.6 m depth, 8 Oct 1964; WAM P. 9131-001, 119+ mm PCL (rostrum damaged), Shark Bay, 25°21’ S, 113°44’ E, 14.6 m depth, 8 Oct 1964; WAM P. 12086-001, 101 mm PCL, Shark Bay, 25°21’ S, 113°44’ E, 10 Mar 1965; WAM P. 14189-001, 117 mm PCL, Shark Bay, 25°21’ S, 113°44’ E, 27 Feb 1962; WAM P. 14664-001, 122 mm PCL, Cape Peron, Shark Bay, 25°30’ S, 113°31’ E, 31 May 1962; WAM P. 14665-001, 65 mm PCL, Hamelin Pool, Shark Bay, 25°21’ S, 113°55’ E, 10 Mar 1961; WAM P. 15522-001, 58 mm PCL, Preston Point, 32°53’ S, 115°39’ E, 18 Nov 1966; WAM P. 19182-001, 19183-001, 19184-001, 90–100 mm PCL, Cockburn Sound, Rockingham, 32°14’ S, 115°46’ E, 26 Feb 1970; WAM P. 23103-001, 98 mm PCL, Swan River, Fremantle, 32°03’ S, 115°44’ E, 13 May 1973; WAM P. 32483-001, 96 mm PCL, Learmonth, 22°15’ S, 114°05’ E, Jun 1973; WAM P. 23859-001, 104 mm PCL, Sunday Island, Exmouth Gulf, 20°05’ S, 114°15’ E, 12.8–18.2 m depth, Aug 1973; WAM P. 25095-001 (2 specimens), 107–111 mm PCL, Exmouth Gulf, 22°05’ S, 114°15’ E, 0–12 m depth, Oct 1974; WAM P. 25348-003, 71 mm PCL, Blackwall Reach, Swan River, 32°02’ S, 115°48’ E, 29 Jun 1975; WAM P. 25352-009, 96 mm PCL, Garden Island, Cockburn Sound, 32°15’ S, 115°38’ E, 3 Jul 1975; WAM P. 25353-004, 94 mm PCL, centre of Cockburn Sound, 32°13’ S, 115°43’ E, 8 Jul 1975; WAM P. 25949-001, 55 mm PCL, Cockburn Sound, 32°11’ S, 115°43’ E, 2 Oct 1977; WAM P. 27219-008, 105 mm PCL, Hummock Island, Houtman Abrolhos Islands, 28°48’ S, 114°03’ E, 40–44 m depth, 22 Nov 1980; WAM P. 27876-001, 40 mm PCL, north of Monkey Mia, Shark Bay, 25°21’ S, 113°44’ E, 1 Dec 1982; WAM P. 27994-001, damaged, Shark Bay, 25°21’ S, 113°44’ E, Dec 1974; WAM P. 29855-001, 58 mm PCL, Koolan Island, Collier Bay, 15°40’ S, 124°10’ E, Jul 1969; WAM P. 30239-052, 78 mm PCL, Exmouth Gulf, 22°15’ S, 114°20’ E, 24 Mar 1991; WAM P. 30690-003, 79 mm PCL, Monte Bello Islands, 20°24’19” S, 115°33’57” E, 8–15 m depth, 23 Aug 1993; WAM P. 31089-006, 58 mm PCL, anchorage west of West Governor Island, Napier Broome Bay, 14°13’ S, 126°35’ E, 25 Nov 1995; WAM P. 31823-002, 26 mm PCL, Norbill Bay, Rosemary Island, Dampier Archipelago, 20°29’ S, 116°36’ E, 3 Aug 2000; WAM P. 31846-005, 103 mm PCL, 5 nm southeast of Sloping Point, Burrup Peninsula, Dampier Archipelago, 20°35.67’ S, 116°54.97’ E, 10–10.5 m depth, 16 Jul 1999; WAM P. 31849-001, 98 mm PCL, 6.2 nm east of Sloping Point, Burrup Peninsula, Dampier Archipelago, 20°32.25’ S, 116°58.48’ E, 16.4–18 m depth, 16 Jul 1999; WAM P. 31864-003, 105 mm PCL, 1.65 nm west-southwest Bluff Point, Enderby Island, Dampier Archipelago, 20°37.74’ S, 116°31.05’ E, 10.5–11 m depth, 19 Jul 1999; WAM P. 32279-003, 127mm PCL, 13.44 km northwest of Cape Peron, Shark Bay, 25°23.122’ S, 116°31.05’ E, 16.8–16.9 m depth, 3 Oct 2002; WAM P. 33177-001 (2 specimens), 106 mm PCL, Jurien Bay, 30°21.03’ S, 114°53.85’ E, 42–43 m depth, 24 Apr 2007.
