# Supplementary material for: Integrated Taxonomy Reveals Hidden Diversity in Northern Australian Fishes: A New Species of Seamoth (Genus Pegasus)
Source: PLoS One. 2016 Mar 2;11(3):e0149415. doi: 10.1371/journal.pone.0149415 (PMC4774964; doi:10.1371/journal.pone.0149415)
Supplement: S2 Text — DNA extractions and sequencing analyses (DOCX) [file pone.0149415.s002.docx]

**S2 Text. DNA barcoding.** DNA extractions and sequencing analyses

DNA from muscle samples were extracted using the Wizard® SV Genomic DNA Purification system (Promega, Australia) with starting material of 0.25g. Tissue extractions were undertaken using SV mini-columns following manufacturer’s instructions (including an overnight digestion at 55°C on an Eppendorf Thermomixer Comfort (Eppendorf, Australia) and the modification of 400µg Proteinase K and DNA were individually precipitated in 160µl nuclease free water. Each DNA sample was quantified on a Nanodrop 8000 UV-Vis Spectrophotometer (Thermo Scientific, USA).

Species identification through barcoding of the 16S and COI mtDNA genes using the universal 16SarL and 16SbrH [1] and FishF1&F2 and FishR2 primers (Ward et al. 2005) respectively were undertaken. PCRs were undertaken in 25µl using GoTaq® Green Master Mix (Promega, USA), Bovine Serum Albumin (Promega, USA), 10µM primers and DNA quantities of between 10 and 15ng. PCRs were performed in an Applied Biosystems GeneAmp® PCR System 9700 (Life Technologies, Thermo Fisher Scientific, USA) with cycling conditions of 94°C × 3min; 35 cycles of 94°C × 1min, 54°C × 1min 30sec, 72°C × 1min; and a final extension of 72°C × 10min. PCR products were visualised on 2.5% TAE agarose gels and fragments cleaned using an Agencourt AMPure XP PCR purification kit (Beckman Coulter, Australia) according to the manufacturer’s instructions. PCR products were sequenced bi-directionally using the same primers as in the original PCR, BigDye® Terminator v3.1 Cycle sequencing kit (Life Technologies) and an annealing stage of 54°C × 5sec across 25 cycles. Cycle sequenced products were cleaned using the CleanSEQ kit (Beckman Coulter) according to the manufacturer’s instructions and run on an ABI 3130XL AutoDNA sequencer (Life Technologies). Remaining archival DNA is stored at -80°C at the CSIRO Marine Laboratories. The 24 consensus sequences per fragments generated here were deposited in GenBank under the following accession numbers (KP796190 – KP796213 (16S sequences) and KP796214 - KP796237 (COI sequences)).

Raw forward and reverse sequences (per gene fragment) were analysed in the first instance in Geneious® R8.1.4 (Biomatters Ltd Auckland, New Zealand; <http://www.geneious.com>). Consensus sequences from each sample were aligned within Geneious using the MUSCLE algorithm and aligned with additional sequences mined from GenBank (AY538972, *P. volitans* 16S large subunit ribosomal RNA gene, partial sequence, Smith and Wheeler (direct submission, American Museum of Natural History, 2004); AP005984, *P. volitans* mitochondrial DNA, complete genome [2]; AP005983, *E. draconis* mitochondrial DNA, complete genome, except for D-loop [2]). Sequence identity was confirmed by using the BLAST module in Geneious (<http://blast.ncbi.nlm.nih.gov/Blast.cgi;Megablast>) against GenBank (<http://www.ncbi.nlm.nih.gov/genbank/>) and comparisons to sequences publicly available in BOLD (<http://www.barcodinglife.com/index.php/IDS_OpenIdEngine>).

Molecular diversity for each mtDNA regions, including average nucleotide composition per putative species (as calculated in MEGA ver. 6.06 [3]) was compared within and among the putative species to evaluate the suitability of the two regions for species identification and to confirm, alongside the alpha taxonomy, the new species status. While this is not a phylogenetic study, prior to phenetic/tree visualisation of the species sequences, the best fit nucleotide evolutionary model for each gene was tested, based on maximum-likelihood fitting in MEGA for each gene. For 16S, the best fit model was Kimura 2-parameter (K2; [4]) while for COI, the model with the lowest Bayesian Information Criterion (BIC) was K2+I. Alongside the sequences mined from GenBank, phenetic analyses were undertaken in MEGA and Geneious and several tree building algorithms including neighbour joining, maximum likelihood (ML) and Bayesian analyses were compared. The three algorithms resulted in highly comparable trees (generated in MEGA ver. 6.06); only the ML analyses are shown here.

**References**

1. Palumbi, S., Martin, A., Romano, S., McMillan, W.O., Stice, L. & Grabowski, G. 2002. The simple fools guide to PCR version 2.0. Honolulu, HI. Department of Zoology and Kewalo Marine Laboratory, University of Hawaii.
2. Kawahara, R., Miya, M., Mabuchi, K., Lavoue, S., Inoue, J.G., Satoh, T.P., Kawaguchi, A., Nishida, M. 2008. Interrelationships of the 11 gasterosteiform families (sticklebacks, pipefishes, and their relatives): a new perspective based on whole mitogenome sequences from 75 higher teleosts. Molecular Phylogenetics and Evolution 46, 224-236.
3. Tamura, K., Stecher, G., Peterson, D., Filipski, A., Kumar, S. 2013. MEGA6: Molecular Evolutionary Genetics Analysis Version 6.0. Molecular Biology and Evolution 30, 2725-2729.
4. Kimura, M. 1980. A simple method for estimating evolutionary rate of base substitutions through comparative studies of nucleotide sequences. Journal of Molecular Evolution 16, 111-120.
