# Supplementary figures and images for: Integrated Taxonomy Reveals Hidden Diversity in Northern Australian Fishes: A New Species of Seamoth (Genus Pegasus)
Source: PLoS One. 2016 Mar 2;11(3):e0149415. doi: 10.1371/journal.pone.0149415 (PMC4774964; doi:10.1371/journal.pone.0149415)

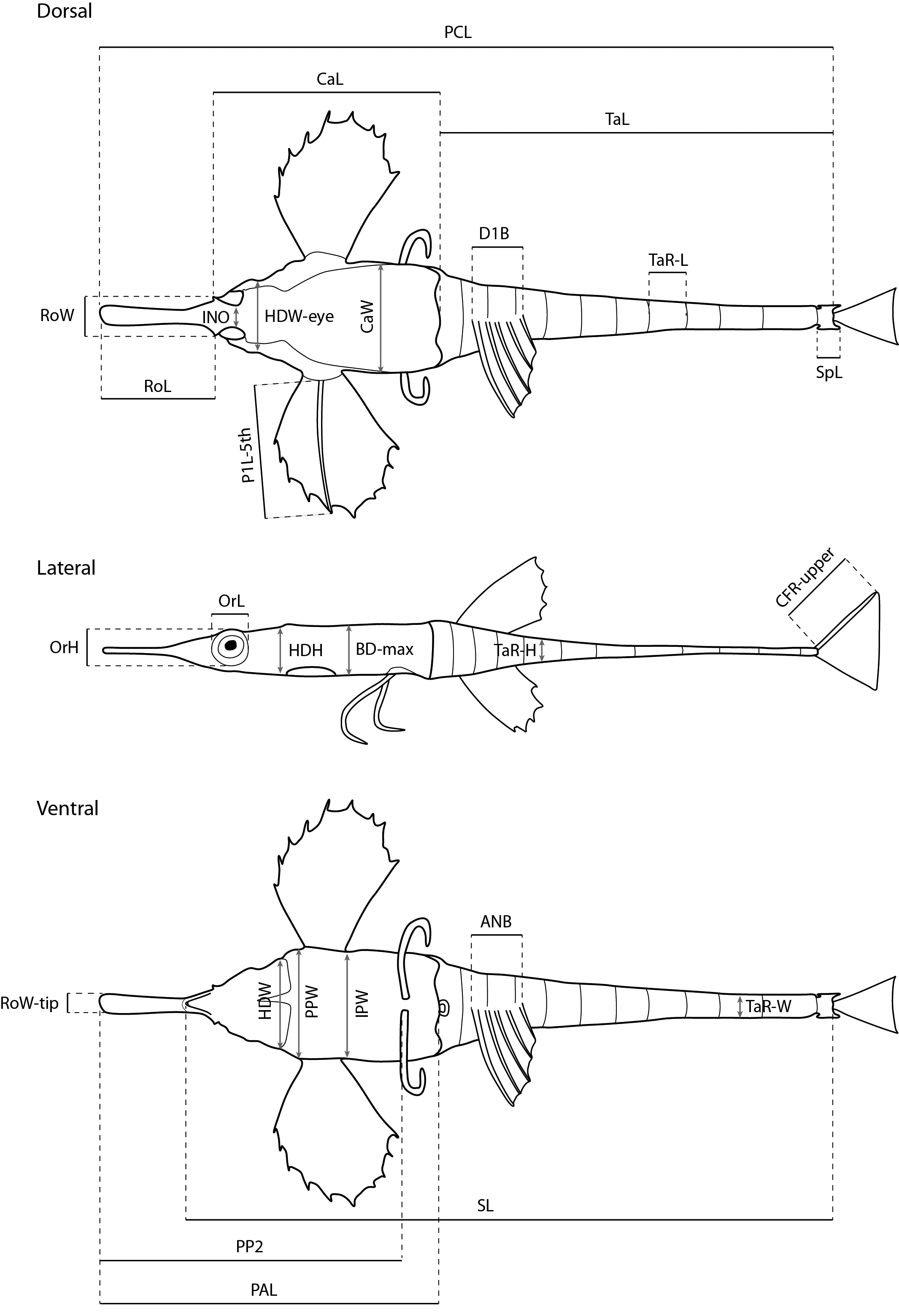

Supplement: S1 Fig — Dorsal, lateral and ventral illustrations of a stylised Pegasus volitans illustrating the measurements used in this study. (TIF) [file pone.0149415.s003.tif]

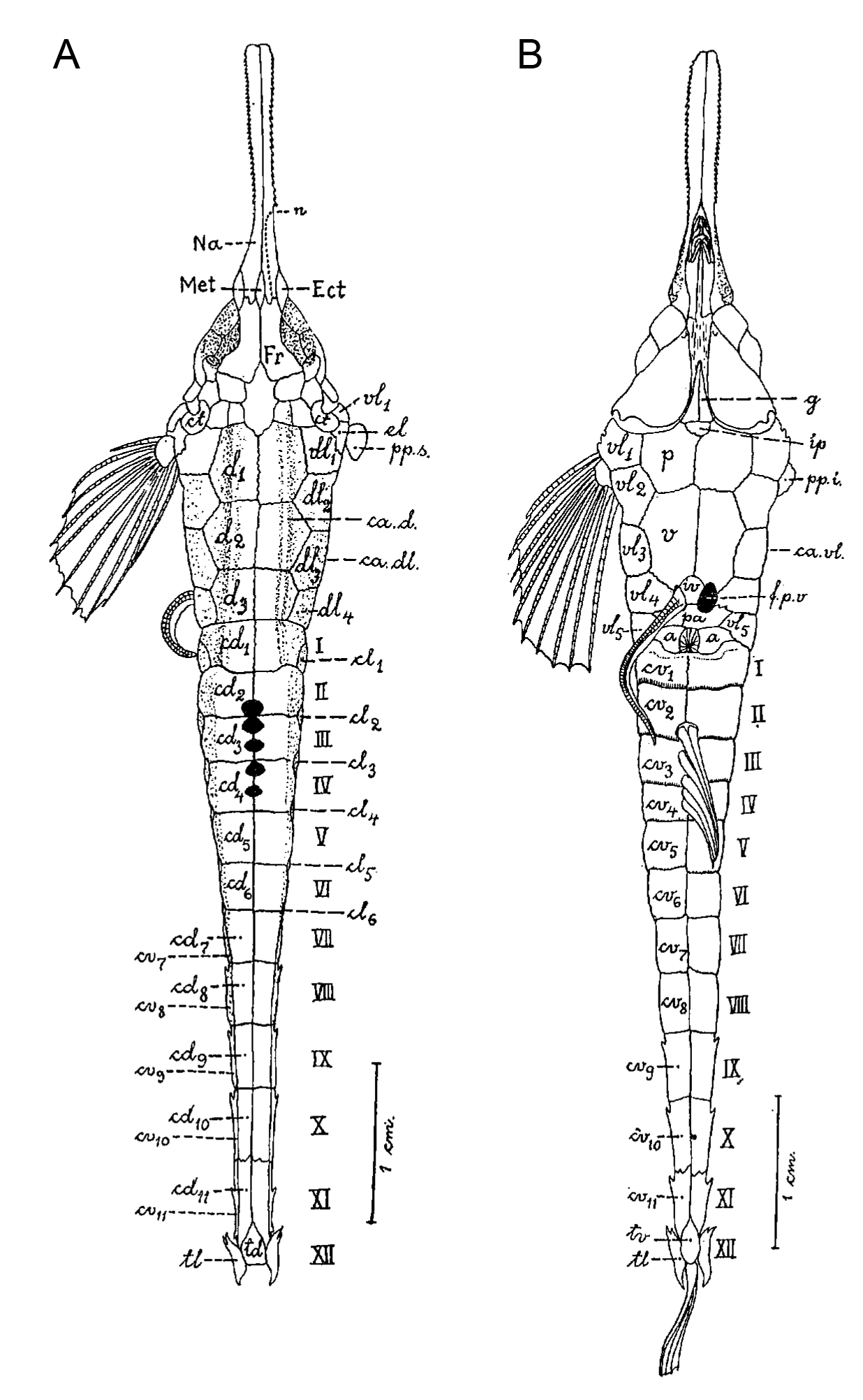

Supplement: S2 Fig — (A) dorsal and (B) ventral views taken from [37]. Abbreviations for head, body and tail plates (all plates are paired except where noted) taken from [14]: a = anal plate; ca.d. = dorsal ridge; ca.dl. = dorsolateral ridge; ca.vl. = ventrolateral ridge; cd1–11 = caudodorsal plates; cll–6 = caudolateral plates; ct = cleithrum; cv1–11 = caudoventral plates; d1–3 = dorsal plates; dll–4 = dorsolateral plates; ect = ectethmoid; f.p.v. = ventral-fin foramen; fr = frontal; g = gular plate; ip = interpectoral plate (unpaired); iv = interventral plate (unpaired); met = mesethmoid (unpaired); na = nasal (paired elements fused on midline); p = pectoral plate; pa = preanal plate (unpaired); pp.s. = superior pectoral-fin plate; pp.i. = inferior pectoral-fin plate; td = terminal-dorsal plate (unpaired); tl = terminal-lateral plate; tv = terminal-ventral plate (unpaired); v = ventral plate; vll–5 = ventrolateral plates. (TIF) [file pone.0149415.s004.tif]
